# Estrogen Regulates Bone Turnover by Targeting RANKL Expression in Bone Lining Cells

**DOI:** 10.1038/s41598-017-06614-0

**Published:** 2017-07-25

**Authors:** Carmen Streicher, Alexandra Heyny, Olena Andrukhova, Barbara Haigl, Svetlana Slavic, Christiane Schüler, Karoline Kollmann, Ingrid Kantner, Veronika Sexl, Miriam Kleiter, Lorenz C. Hofbauer, Paul J. Kostenuik, Reinhold G. Erben

**Affiliations:** 1 Department of Biomedical Research, University of Veterinary Medicine Vienna, Vienna, Austria; 2 Department for Companion Animals and Horses, University of Veterinary Medicine Vienna, Vienna, Austria; 3 Division of Endocrinology, Diabetes, and Bone Diseases, Department of Medicine III and Center for Healthy Aging, Technische Universität Dresden, Dresden, Germany; 4 Amgen Inc., Thousand Oaks, CA USA; 5 Phylon Pharma Services, Newbury Park, CA USA; 6 UCB Pharma GmbH, Vienna, Austria

## Abstract

Estrogen is critical for skeletal homeostasis and regulates bone remodeling, in part, by modulating the expression of receptor activator of NF-κB ligand (RANKL), an essential cytokine for bone resorption by osteoclasts. RANKL can be produced by a variety of hematopoietic (e.g. T and B-cell) and mesenchymal (osteoblast lineage, chondrocyte) cell types. The cellular mechanisms by which estrogen acts on bone are still a matter of controversy. By using murine reconstitution models that allow for selective deletion of estrogen receptor-alpha (ERα) or selective inhibition of RANKL in hematopoietic vs. mesenchymal cells, in conjunction with *in situ* expression profiling in bone cells, we identified bone lining cells as important gatekeepers of estrogen-controlled bone resorption. Our data indicate that the increase in bone resorption observed in states of estrogen deficiency in mice is mainly caused by lack of ERα-mediated suppression of RANKL expression in bone lining cells.

## Introduction

Estrogen is an important regulator of bone mass. The role of estrogen for bone homeostasis in humans is illustrated by the fact that estrogen deficiency is one of the major causes of postmenopausal osteoporosis^1^. Estrogen acts through two receptors, estrogen receptor-alpha (ERα) and -beta (ERβ), with ERα being more important for the regulation of bone metabolism^2^. Estrogen receptors are widely expressed in a variety of cells in bone and bone marrow. However, the actual target cell responsible for mediating the effects of estrogen on bone is still a matter of debate^3^.

One of the most important downstream mediators of the action of estrogen on bone is the osteoprotegerin (OPG)/receptor activator of NF-κB ligand (RANKL) system. RANKL is an essential cytokine for osteoclast differentiation, activation, and survival^4, 5^. RANKL is produced by a variety of cells such as cells of the stromal cell lineage, activated T lymphocytes, but also B lymphocytes^5^. OPG is a soluble decoy receptor for RANKL which binds RANKL, and thereby inhibits osteoclastogenesis^6^. RANKL acts through the receptor RANK which is expressed in the cell membrane of osteoclasts and osteoclast precursor cells^7^. RANKL, RANK, and OPG are essential, non-redundant factors for osteoclast biology. Osteoclasts are entirely absent in RANK or RANKL deficient mice, leading to osteopetrosis, whereas OPG-deficient mice exhibit excessive bone resorption and severe osteoporosis^5, 7, 8^. RANKL exists in two biologically active forms, a membrane-bound form and a soluble form. Membrane-bound RANKL can be shed by matrix metalloproteinase 14 (MMP-14) or by a disintegrin and metalloproteinase (ADAM) 10^9^ resulting in soluble RANKL. In addition, soluble RANKL is produced by immune cells as a primary secreted form^5^.

It is well established that sex steroids regulate the RANKL-OPG axis in osteoblast-like cells *in vitro* ^10, 11^. There is good evidence that OPG is regulated directly by sex hormones, whereas the sex steroid-mediated regulation of RANKL appears to be mainly indirect^10–13^. *In vivo*, ovariectomy (OVX) of female rats and orchiectomy (ORX) of male rats increased RANKL mRNA expression in bone^14, 15^. Moreover, free soluble RANKL was found to be increased in bone marrow plasma of male ORX rats^16, 17^. However, the specific cellular origin of RANKL in the sex steroid deficiency models remained undefined. The importance of RANKL in the pathogenesis of estrogen deficiency-induced bone loss can be further deduced from the fact that antibodies directed against RANKL completely inhibit bone loss in ovariectomized mice^18^ and in women with postmenopausal osteoporosis^19^. However, the target cell(s) which translate the effects of sex steroids to bone by altering their expression of RANKL and/or OPG remained undefined.

Conditional ablation of ERα in cells of the osteoblastic lineage (osteoblast progenitors, osteoblasts, osteocytes) has been reported to induce bone loss or to compromise bone accrual in female mice^20–23^. In addition, conditional ablation of RANKL from osteocytes revealed an important role of osteocyte-derived RANKL for the control of osteoclast formation and bone homeostasis^24–27^. Moreover, it was shown that estrogen protects bone by regulating the survival of osteoclasts through upregulated secretion of TGFß^28^, and of the apoptosis-promoting Fas ligand in osteoblasts^29^. These studies point at the increased expression of RANKL and/or at the suppression of paracrine osteoclast apoptosis signals in cells of the osteoblastic lineage as the main mechanism underlying the upregulation of bone turnover upon estrogen withdrawal.

On the other hand, there is strong experimental evidence suggesting that estrogen regulates bone turnover by targeting cells of the hematopoietic lineage, i.e. immune cells or osteoclasts. The conditional ablation of ERα in osteoclasts was shown to protect against OVX-induced bone loss^30, 31^. Further studies in mice led to the hypothesis that T lymphocytes mediate the effects of sex steroid deficiency on bone turnover^32, 33^. Similarly, B lymphocytes were proposed to play a role as relevant source of osteoclastogenic cytokines after sex steroid withdrawal. In this context, it was reported that OVX upregulated RANKL mRNA expression in B220^+^ B cells, but not in adherent marrow stromal cells^34^. In line with these data, conditional ablation of RANKL in B lymphocytes partially protected against OVX-induced bone loss^35^. A flow cytometric analysis of human bone marrow aspirates confirmed that estrogen deficiency in women is associated with an up-regulation of membrane-bound expression of RANKL on osteoblastic cells, T cells and B cells^36^.

Taken together, there is solid evidence that sex steroids regulate the RANKL-OPG axis in the bone microenvironment, whereas the relative contribution of immune cell-derived versus mesenchymal cell-derived RANKL remains poorly defined. To clarify whether hematopoietic or mesenchymal cells are the main effector cells mediating the detrimental effects of estrogen deficiency on bone, we established reconstitution models resulting in selective deletion of ERα or selective inhibition of RANKL in hematopoietic versus mesenchymal cells in mice. The mesenchymal cell compartment was further refined by *in situ* mRNA expression profiling, using laser capture microdissection. Here, we report that estrogen regulates bone metabolism by primarily targeting RANKL expression in bone lining cells. Bone lining cells are osteoblast-derived cells which cover all quiescent bone surfaces.

## Results

### Lethal irradiation followed by reconstitution with unfractionated bone marrow reconstitutes the hematopoietic but not the mesenchymal cell compartment

To establish a robust reconstitution model that allows for a nearly complete replacement of the hematopoietic compartment, we employed transgenic mice on the C57BL/6 genetic background that ubiquitously express the marker gene human placental alkaline phosphatase (hPLAP) under the control of a ROSA26 promoter^37^. hPLAP is expressed in the cell membrane, and is readily detected by flow cytometry, histochemistry, and immunohistochemistry^38, 39^. Upon a single lethal irradiation dose of 10 Gy, transplantation of unfractionated bone marrow cells derived from hPLAP transgenic mice efficiently reconstituted the hematopoietic system with a chimerism (ratio of hPLAP-positive donor-derived vs. hPLAP-negative recipient-derived cells) greater than 90% as analyzed by flow cytometry, 4 weeks post-transplantation (Suppl. Fig. 1A and B). All subpopulations in bone marrow were completely reconstituted, 4 weeks post-transplantation (Suppl. Fig. 1C). Raising the irradiation dose to 11 and 12 Gy did not significantly improve the experimental system and only resulted in minimal further increases in bone marrow chimerism (Suppl. Fig. 1A). Thus, we used a single dose of 10 Gy for all subsequent irradiation experiments. In contrast to the hematopoietic compartment, which includes osteoclasts, mesenchymal stem cells isolated from bones of reconstituted mice remained hPLAP-negative and thus exclusively recipient-derived, both 4 and 16 weeks post-transplantation (Suppl. Fig. 1D). This finding is in clear agreement with an earlier study in bone marrow-transplanted rats^40^. Both studies show that mesenchymal precursor cells fail to engraft after lethal irradiation and subsequent bone marrow transplantation with unfractionated bone marrow, probably because there is no niche void in the host due to the greater resistance of the stromal cell compartment to irradiation^40^. The life span of mature murine osteoclasts is assumed to be in the range of three days^41^. Therefore, osteoclasts surviving lethal irradiation can be ruled out as a possible confounder, 4 weeks post-transplantation. Complete separation between the donor-derived hematopoietic compartment and the recipient-derived mesenchymal compartment in reconstituted mice provided a unique and powerful opportunity to exploit this system to pursue an unbiased approach for identifying the estrogen target cell lineage in bone.

### Lethal irradiation and subsequent bone marrow transplantation are not directly associated with bone loss in estrogen-replete mice

**Figure 1:**
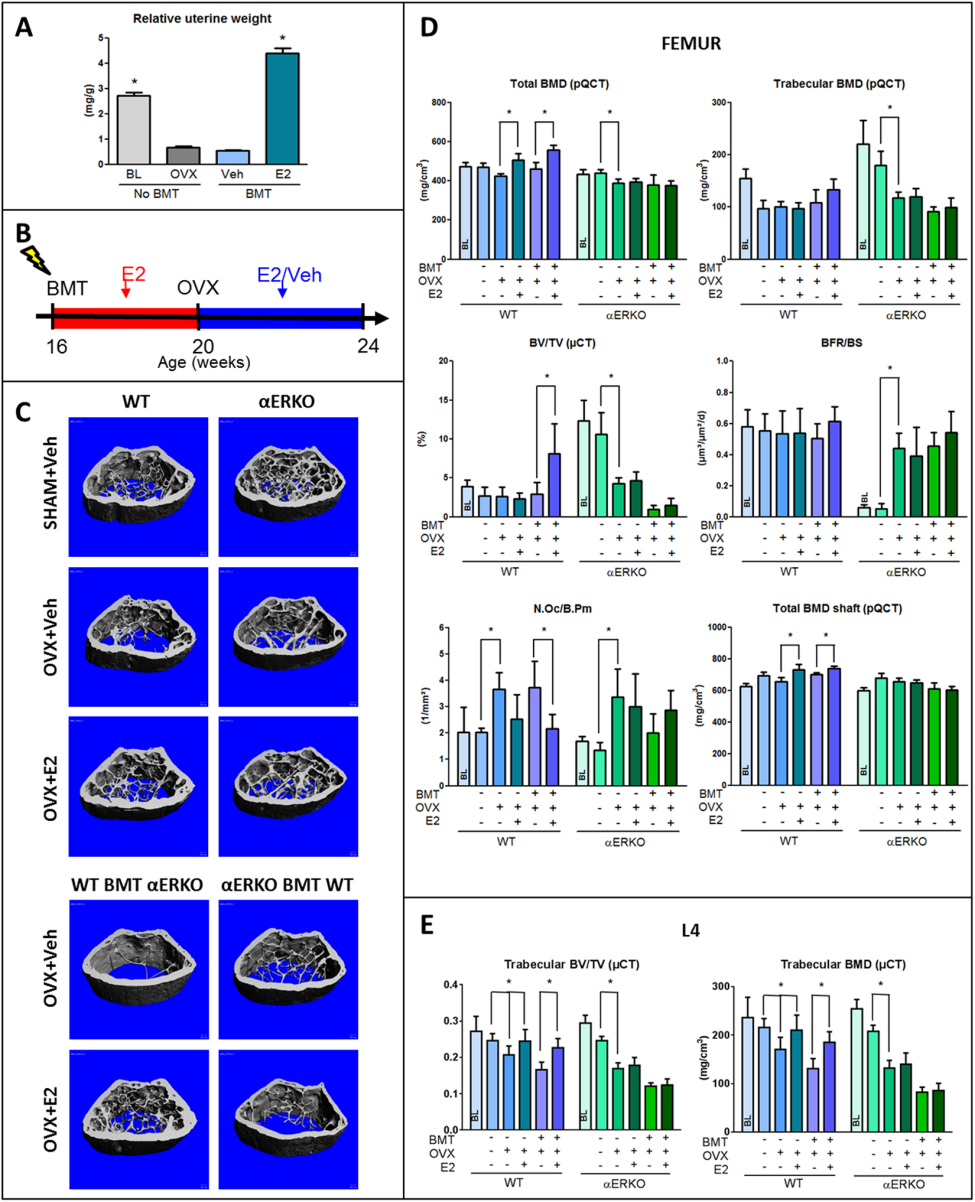
Selective deletion of estrogen receptor α in the mesenchymal or hematopoietic compartment. (**A**) Relative to baseline (BL) controls, uterine weight decreased in non-irradiated (No BMT) WT mice 4 weeks after OVX, similar to the decrease seen 4 weeks after irradiation (BMT) of vehicle-treated ovary-intact WT mice (Veh); 17ß-estradiol (E2) treatment of irradiated WT mice significantly increased uterine weight. (**B**) Experimental design. All irradiated mice were supplemented with physiological doses of E2 (10 µg/kg s.c. in B/R 5 times per week) during the 4-week recovery phase post-transplantation. (**C**) µCT images of the femoral distal metaphysis of non-irradiated (upper panels) SHAM and vehicle- or 17ß-estradiol (E2)-treated OVX WT and αERKO mice, and vehicle- or estradiol-treated WT and αERKO mice transplanted (BMT, lower panels) with unfractionated bone marrow cells from αERKO or WT mice, 4 weeks post-OVX. (**D**) Total and trabecular BMD measured by pQCT and bone volume (BV/TV) measured by µCT in the distal femoral metaphysis, bone formation rate (BFR/BS) and osteoclast number (N.Oc/B.Pm) in cancellous bone of the distal femoral metaphysis measured by histomorphometry, total BMD of the femoral shaft measured by pQCT, and (**E**) bone volume (BV/TV) as well as trabecular BMD of the L4 vertebrae in non-irradiated and reconstituted WT and αERKO mice treated with vehicle or physiological doses of E2, 4 weeks post-OVX. All irradiated mice were supplemented with physiological doses of E2 during the 4-week recovery phase post-transplantation. Data represent mean ± SD of 8–10 animals each. *p < 0.05 by one-way ANOVA followed by SNK test.

In order to exclude that irradiation *per se* causes bone loss, C57BL/6 wild-type (WT) bone marrow was transplanted into lethally irradiated C57BL/6 WT mice that were 16 weeks old, an age when longitudinal bone growth has largely ceased. As expected, irradiation caused uterine atrophy, to a similar extent as that inflicted by ovariectomy (Fig. 1A). This is presumably due to the sensitivity of oocytes to whole body irradiation^42^, that can lead to ovarian failure and subsequent estrogen deficiency. A study conducted in non-irradiated OVX C57BL/6 mice identified an estradiol dosing regimen (10 µg/kg, formulated in ricinus oil/benzyl benzoate, injected 5 times/week s.c.) that conferred near-physiological estrogen replacement to the uterus and bone (Suppl. Fig. 2A and B), and this regimen was then applied to irradiated C57BL/6 mice to negate the indirect effects of irradiation on bone. This dose of estradiol results in slight over-supplementation in irradiated mice at the uterus (Fig. 1A and Suppl. Fig. 2D) and at the distal femur (Suppl. Fig. 2B). However, a dose of 10 µg/kg was necessary to fully antagonize post-OVX bone loss at the spine (Suppl. Fig. 2B), and this dose was used in all subsequent experiments.

The irradiation-induced destruction of ovarian oocytes necessitated the experimental design shown in the scheme in Fig. 1B. Briefly, in the 4-week reconstitution phase after lethal irradiation and bone marrow transplantation, all mice were supplemented with estradiol. Thereafter, the mice were ovariectomized, and received vehicle or estradiol during the 4-week experimental period. Age-matched non-irradiated SHAM + vehicle, OVX + vehicle, and OVX + estradiol groups served as controls. Irradiation was not associated with loss of body weight (Suppl. Fig. 2D). Both vehicle- and estradiol-treated irradiated and non-irradiated OVX mice showed a similar femoral cancellous bone structure as evidenced by µCT analysis (Suppl. Fig. 2C) and an identical femoral bone mineral density (BMD) measured by peripheral quantitative computed tomography (pQCT) (Suppl. Fig. 2E), indicating that irradiation *per se* did not have a detrimental effect on femoral bone mass. Interestingly, estradiol exerted an anabolic effect in the cancellous bone of the distal femur in irradiated, relative to non-irradiated mice (Suppl. Fig. 2C and E), an effect not observed at the spine (Suppl. Fig. 2E). The underlying reason for this phenomenon is unclear but may be related to residual bone elongation occurring during the post-transplantation period at the distal femoral growth plate. pQCT analysis of the spine did not show differences in BMD between vehicle- and estradiol-treated irradiated and non-irradiated OVX mice (Suppl. Fig. 2E). However, µCT analysis of L4 lumbar vertebral bodies revealed a small, but significant reduction in cancellous bone volume in irradiated vehicle-treated, but not estradiol-treated OVX mice, relative to non-irradiated controls (Suppl. Fig. 2E). In summary, our data show that whole body irradiation with a single dose of 10 Gy lacks negative effects on femoral and vertebral bone mass or structure in estrogen-replete mice, and leads to an only minor decrease in vertebral cancellous bone volume in estrogen deficient mice.

### Selective deletion of ERα in mesenchymal but not hematopoietic cells determines estrogen response in bone

To address whether hematopoietic (e.g. lymphocytes, osteoclasts) or mesenchymal (e.g. mesenchymal stem cells, osteoblastic lineage, chondrocytes, adipocytes) cells are the main effector cells responsible for mediating the effects of estrogen on bone, we established a reconstitution model resulting in selective deletion of ERα in hematopoietic or mesenchymal cells. To this end, we lethally irradiated 16-week-old female WT and global ERα knockout mice (αERKO) on C57BL/6 genetic background, and reconstituted them with sex-matched bone marrow from αERKO or WT mice, respectively. In irradiated WT mice reconstituted with αERKO mouse bone marrow, all mesenchymal cells are of recipient origin and express a functional ERα, whereas hematopoietic cells are of donor-origin and lack ERα. Conversely, in irradiated αERKO mice reconstituted with WT bone marrow, hematopoietic cells express a functional ERα, whereas mesenchymal cells lack ERα. To control for the aforementioned effects of irradiation on ovarian function, irradiated mice received physiological doses of 17β-estradiol (10 µg/kg) 5 times per week over 4 weeks post-irradiation (see scheme in Fig. 1B). Thereafter, all mice were ovariectomized, and received either vehicle or 10 µg/kg 17β-estradiol (s.c.) 5 times per week. Non-irradiated sham-operated, OVX and estradiol-supplemented OVX WT and αERKO mice served as controls.

As depicted in Fig. 1C and D total distal femoral volumetric BMD was reduced in non-irradiated vehicle-treated WT and αERKO OVX mice relative to baseline, 1 month post-OVX. Ovariectomy did not cause cancellous bone loss in distal femurs of non-irradiated WT mice as evidenced by pQCT and µCT analysis (Fig. 1C and D), suggesting that ovariectomy-induced femoral bone loss in 4-month-old, non-growing C57BL/6 mice mainly occurs at endocortical bone surfaces which is also evident from the µCT images shown in Fig. 1C. In contrast, ovariectomy caused cancellous bone loss in lumbar vertebrae of non-irradiated mice, which was prevented by estrogen replacement therapy (Fig. 1E). It was reported earlier that αERKO mice are characterized by a high bone mass phenotype which is caused by high circulating androgen and estrogen levels due to lacking ERα-mediated hypothalamic negative feedback control^2^. It is thought that the increased ovarian production of androgens is the main reason for the high bone mass phenotype observed in these mice, because anti-androgen, but not anti-estrogen treatment recapitulated the effect of ovariectomy in female αERKO mice^2^. Accordingly, αERKO mice lost femoral and vertebral cancellous bone after ovariectomy (Fig. 1C–E and Suppl. Fig. 3), corroborating earlier reports^2^. As expected, bone and uterus of OVX αERKO mice did not respond to physiological doses of estradiol (Fig. 1C–E and Suppl. Fig. 4).

Similar to non-irradiated OVX WT mice, estrogen treatment of OVX WT mice reconstituted with αERKO bone marrow increased bone mass at the femoral metaphysis, the femoral shaft, and the lumbar spine, relative to vehicle-treated OVX controls (Fig. 1C–E). In contrast, estrogen treatment failed to increase bone mass in OVX αERKO mice reconstituted with WT mouse bone marrow (Fig. 1C–E). Cancellous bone formation rate remained unchanged by ovariectomy in the distal femoral metaphysis of WT mice and WT mice transplanted with αERKO bone marrow, 4 weeks post-ovariectomy. In contrast, the bone formation rate increased after ovariectomy of αERKO mice compared with non-irradiated SHAM αERKO controls, but did not respond to physiological doses of estradiol (Fig. 1D). Osteoclast numbers in the cancellous bone of the distal femoral metaphysis increased after ovariectomy of WT and αERKO mice (Fig. 1D). Estradiol treatment suppressed osteoclast numbers in WT mice and WT mice reconstituted with αERKO bone marrow, but not in αERKO mice or αERKO reconstituted with WT bone marrow (Fig. 1D). In summary, these results unequivocally show that a functional ERα in cells of the mesenchymal cell lineage, but not in hematopoietic cells, mediates the effects of estrogen on bone.

### RANKL derived from mesenchymal but not hematopoietic cells accounts for the regulation of bone turnover

**Figure 2:**
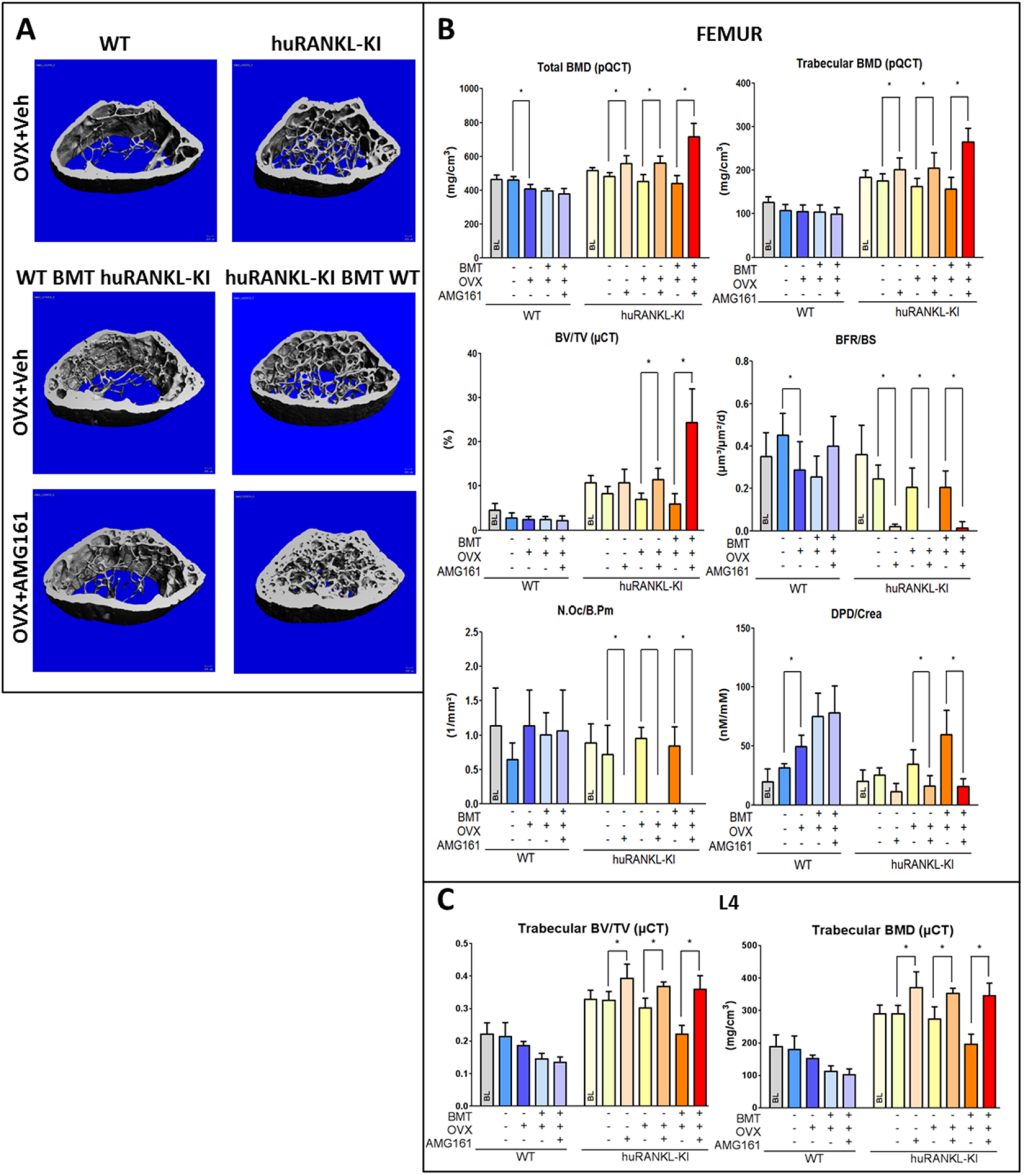
Selective inhibition of RANKL in the hematopoietic or mesenchymal compartment. (**A**) µCT images of the distal femoral metaphysis of non-irradiated vehicle-treated OVX WT and huRANKL-KI mice (upper panels), and vehicle- or AMG161 (10 mg/kg twice weekly)-treated WT and huRANKL-KI mice transplanted (BMT) with unfractionated bone marrow cells from huRANKL-KI or WT mice, 4 weeks post-OVX. (**B**) Total and trabecular BMD measured by pQCT, bone volume (BV/TV) measured by µCT, and bone formation rate (BFR/BS) and osteoclast number (N.Oc/B.Pm) measured by histomorphometry in cancellous bone of the distal femoral metaphysis, urinary deoxypyridinoline/creatinine (DPD/Crea) excretion measured by ELISA, and (**C**) bone volume (BV/TV) as well as trabecular BMD of the L4 vertebrae in non-irradiated and reconstituted WT and huRANKL-KI mice treated with vehicle or AMG161, 4 weeks post-OVX. All irradiated mice were supplemented with physiological doses of E2 during the 4-week recovery phase post-transplantation. Data represent mean ± SD of 8–10 animals each. *Denotes p < 0.05 by one-way ANOVA followed by SNK test.

The availability of novel mice that exclusively express a humanized *rankl* gene, in conjunction with our irradiation-transplant approach, provided the opportunity to address whether the RANKL source that drives estrogen deficiency-induced bone loss is derived from hematopoietic or mesenchymal cells. Human RANKL knock-in (huRANKL-KI) mice carry the human instead of the murine exon 5 in their *rankl* gene, and express a chimeric RANKL protein wherein most of the RANK binding domain is human^43^. The anti-huRANKL antibody AMG161 blocks human and chimeric, but not murine RANKL^43^. The chimeric RANKL protein expressed by huRANKL-KI mice activates murine RANK and is thus capable of inducing bone resorption in mice, while being fully inhibited by human-specific RANKL antibodies^43^. Of note, all isoforms of RANKL (membrane-bound and soluble RANKL) are encoded by one gene, and all cells in homozygous huRANKL-KI mice, including immune cells, produce only chimeric RANKL. Previous studies indicated that huRANKL-KI mice exhibit a high bone mass phenotype^43, 44^ which may suggest slightly reduced biological activity of the chimeric RANKL protein^43^. The greater bone mass in huRANKL-KI mice compared with WT mice is confirmed in our study (Fig. 2A–C).

To establish a model allowing to selectively block hematopoietic or mesenchymal cell-derived RANKL, we lethally irradiated 16-week-old female WT and huRANKL-KI mice on C57BL/6 genetic background. Mice were subsequently reconstituted with sex-matched bone marrow from hRANKL-KI or WT mice, respectively. In irradiated WT mice reconstituted with huRANKL mouse bone marrow, mesenchymal cell-derived RANKL is exclusively murine, whereas hematopoietic cell-derived RANKL is exclusively chimeric and inhibited by AMG161. Conversely, in irradiated huRANKL mice reconstituted with WT bone marrow, hematopoietic cell-derived RANKL is exclusively murine, while mesenchymal cell-derived RANKL is exclusively chimeric and inhibited by AMG161. To control for the effects of irradiation on ovarian function, irradiated mice received physiological doses of 17β-estradiol (10 µg/kg) 5 times per week over 4 weeks post-irradiation. Thereafter, all mice were ovariectomized, and received either physiological saline or AMG161 (10 mg/kg, s.c.) twice weekly. Non-irradiated OVX WT and huRANKL-KI mice treated with saline or AMG161 served as controls. All mice were killed 4 weeks post-OVX. To exclude the possibility that irradiation and reconstitution might alter RANKL expression in transplanted hematopoietic cells, we examined RANKL expression by immunohistochemistry in the spleen. However, there was no difference in splenic RANKL expression between irradiated and non-irradiated mice, ruling out that irradiation and reconstitution alters hematopoietic RANKL expression (Suppl. Fig. 5).

Similar to OVX huRANKL-KI mice treated with AMG161, marked increases in femoral and vertebral bone mass were observed upon AMG161 treatment of OVX huRANKL-KI mice reconstituted with WT bone marrow as evidenced by pQCT and µCT analysis (Fig. 2A–C and Suppl. Fig. 6). In contrast, AMG161 failed to increase bone mass in OVX WT mice reconstituted with huRANKL-KI mouse bone marrow (Fig. 2A–C and Suppl. Fig. 6). AMG161 profoundly suppressed osteoclast numbers, urinary excretion of collagen crosslinks, and bone formation rate in huRANKL-KI mice reconstituted with WT bone marrow mice, expressing chimeric RANKL in mesenchymal cells (Fig. 2B). In contrast, no effects were observed in WT mice reconstituted with huRANKL-KI mouse bone marrow, expressing chimeric RANKL in hematopoietic cells (Fig. 2B). These results demonstrate that RANKL derived from mesenchymal, but not from hematopoietic cells, is relevant for the physiological regulation of bone metabolism in OVX mice.

### Estrogen deficiency selectively enhances RANKL expression in bone lining cells

**Figure 3:**
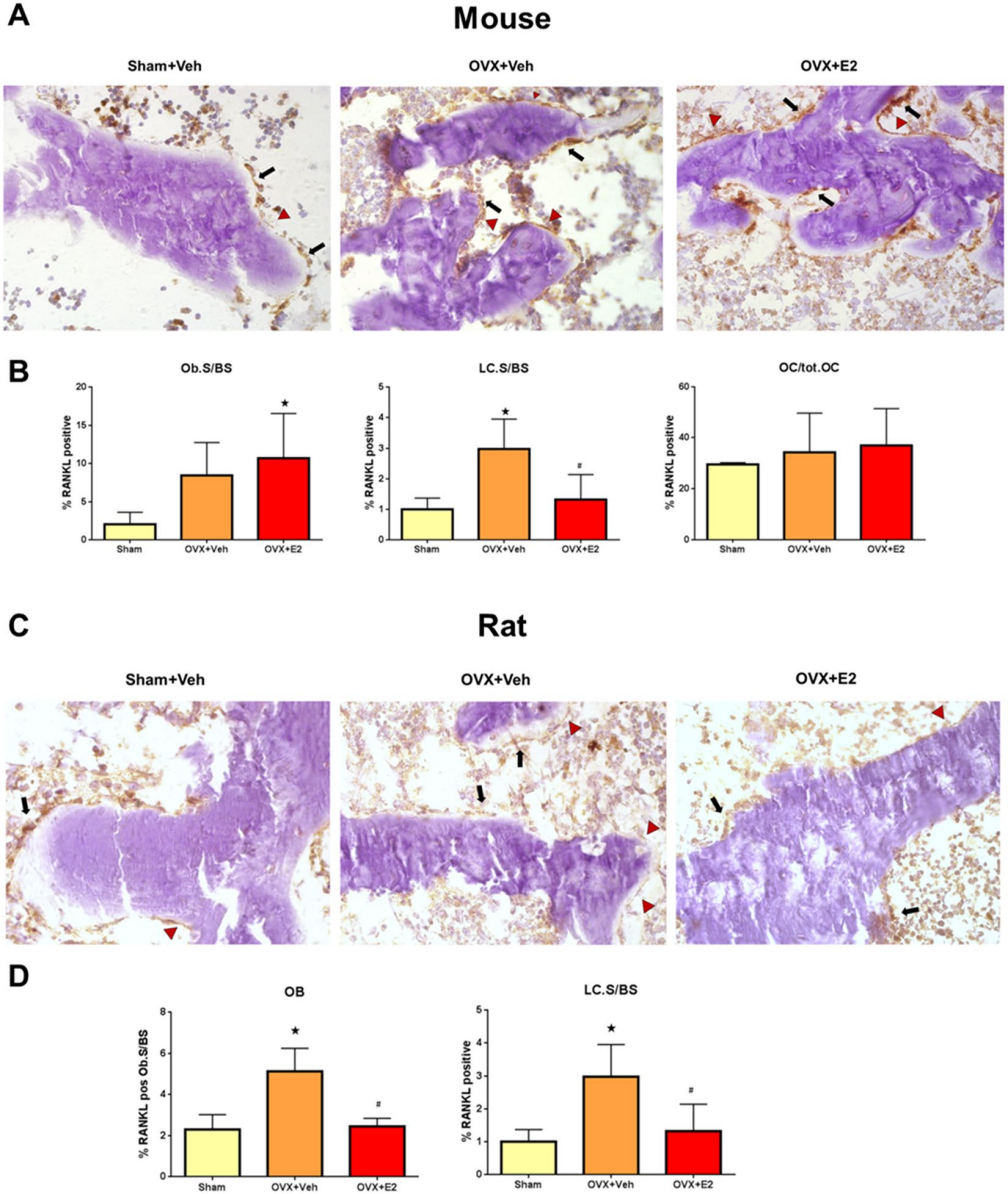
Estradiol targets RANKL expression in bone lining cells. (**A**) Anti-RANKL immunohistochemistry on cryosections of undecalcified distal femurs in vehicle-treated SHAM, and vehicle- or 17ß-estradiol (10 µg/kg in B/R 5 times per week)-treated OVX WT mice, 2 weeks post-OVX. (**B**) Percent RANKL-positive osteoblasts per bone surface (BS), percent RANKL-positive bone lining cells (LC) per bone surface, and percent RANKL-positive osteocytes in vehicle-treated SHAM and vehicle- or E2-treated OVX WT mice, 2 weeks post-OVX. (**C**) Anti-RANKL immunohistochemistry on cryosections of undecalcified proximal tibias in vehicle-treated SHAM and vehicle- or E2 (2.5 µg/kg in B/R 5 times per week)–treated OVX Fischer 344 rats, 2 weeks post-OVX. (**D**) Percent RANKL-positive osteoblasts per bone surface (BS) and percent RANKL-positive bone lining cells per bone surface in vehicle-treated SHAM and vehicle- or E2-treated OVX rats, 2 weeks post-OVX. Arrows in A and C mark RANKL-positive osteoblasts, arrowheads mark RANKL-positive lining cells. *In B and D denotes P < 0.05 vs. Sham, ^#^denotes P < 0.05 vs. OVX + vehicle. Data in B and D are mean ± SD of 4–5 animals each.

So far, our data provide evidence that estradiol regulates bone turnover by targeting RANKL expression in cells in the mesenchymal bone compartment which is comprised of osteocytes, osteoblasts, and bone lining cells. To further identify the estrogen target cell within the mesenchymal cell compartment, we initially performed anti-RANKL immunohistochemistry, 2 weeks post-OVX in mice and rats. This time point was chosen because the increase in bone resorption induced by ovariectomy is maximal at 2 weeks post-OVX^45, 46^. Immunohistochemistry was performed on cryosections of undecalcified bone, using the recently established tape technology^47^. We validated the specificity of the primary anti-RANKL antibody (Santa Cruz) used in this study by comparison with another anti-RANKL antibody (R&D), which was shown to be highly specific by using bone cryosections of *Rankl* ^∆/∆^ mice, which express a truncated form of RANKL^24^ (Suppl. Figs 7 and 8). RANKL protein expression was detectable in only about a third of cancellous bone osteocytes in SHAM and OVX mice, and was not regulated by ovariectomy or estrogen replacement (Fig. 3A and B). In contrast, estrogen deficiency distinctly up-regulated the relative surface extent of RANKL-expressing bone lining cells (Fig. 3A and B). A similar, but non-significant trend was seen in RANKL-expressing osteoblasts (Fig. 3B). Treatment of OVX mice with estradiol suppressed the relative bone surface covered with RANKL-expressing bone lining cells, but not that of RANKL-expressing osteoblasts (Fig. 3A and B).

Unlike higher mammals such as humans, estrogen stimulates osteoblastic bone formation in mice^48^. Considering such differential osteoblast responses to estrogen, we examined whether the RANKL findings in mice were corroborated in OVX rats. Similar to humans, estrogen suppresses both bone formation and bone resorption in rats^48^. Ovariectomy resulted in a ~2-fold increase in the surface extent of RANKL-positive osteoblasts and lining cells, which was reduced to SHAM control levels by estradiol treatment of OVX rats (Fig. 3C and D). In concordance with the mouse experiment, RANKL expression was detectable in only about a third of cancellous bone osteocytes of SHAM and OVX rats, and was not regulated by estrogen deficiency (data not shown). Thus, upregulation of RANKL-expressing bone lining cells was a common feature after OVX of both mice and rats.

**Figure 4:**
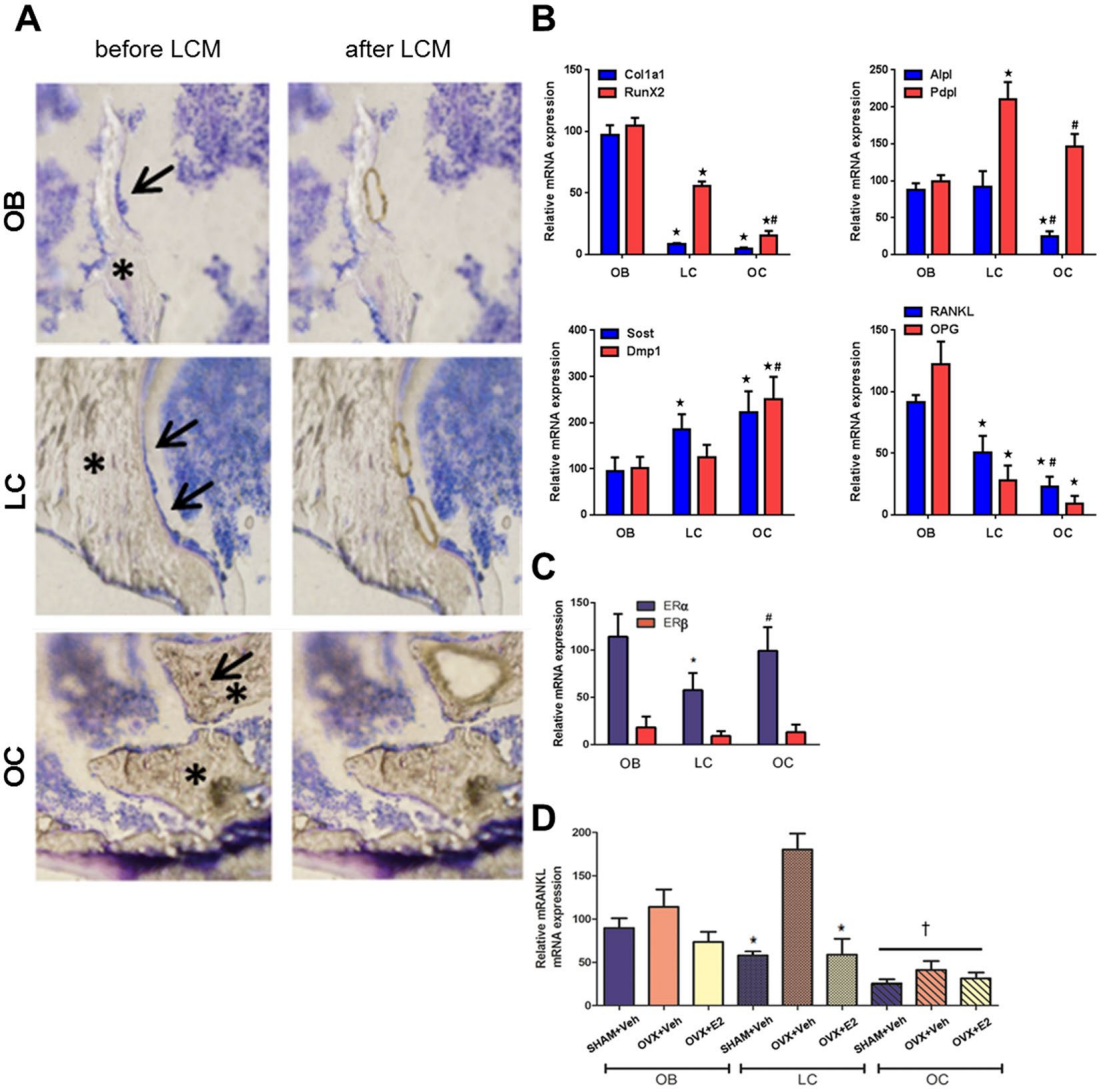
*In situ* mRNA profiling of bone cells in cryosections by laser capture microdissection (LCM). (**A**) Harvesting of osteoblasts (OB), bone lining cells (LC), and osteocytes (OC) by LCM in 4-µm-thick cryosections of mouse femurs. Left panels show sections before LCM with cells to be harvested marked by arrows, right panels show the same section after LCM. Note that the retraction of the bone marrow facilitates harvesting of especially bone lining cells which remain attached to the bone surface. Cryosections were briefly stained with Histostain (Arcturus). Asterisks mark bone tissue. (**B**) mRNA expression profiling of collagen 1a1 (Col1a1), Runx2, alkaline phosphatase (Alpl), Podoplanin (Pdpl), sclerostin (Sost), dentin matrix protein-1 (Dmp1), RANKL, and OPG by qRT-PCR on RNA isolated from distal femoral cancellous bone osteoblasts (OB), osteocytes (OC), and bone lining cells (LC) harvested by LCM in cryosections of undecalcified bones from WT mice. (**C**) mRNA expression of ERα and ß in distal femoral cancellous bone osteoblasts, osteocytes, and bone lining cells harvested by LCM in cryosections of undecalcified bones from WT mice. mRNA expression of ERß was expressed relative to ERα expression in osteoblasts. (**D**) mRNA expression of RANKL in distal femoral cancellous bone osteoblasts, osteocytes, and bone lining cells harvested by LCM in cryosections of undecalcified bones from vehicle-treated SHAM and vehicle- or E2-treated OVX WT mice. Data in B-D represent mean ± SD of 3–5 animals each. In B and C, *denotes P < 0.05 vs. osteoblasts, ^#^denotes P < 0.05 vs. lining cells by one-way ANOVA followed by SNK test. In D, *denotes P < 0.05 vs. OVX + vehicle, ^†^denotes P < 0.05 vs. the same treatment in osteoblasts and lining cells by one-way ANOVA followed by SNK test.

Little is known about the phenotype of bone lining cells *in situ*. We thus established the novel technology of laser capture microdissection (LCM) of undecalcified cryosections from mouse femurs to isolate bone lining cells, using standard morphological criteria to define the different cell types (Fig. 4A). We employed this method recently to analyze gene expression in osteoblasts^49, 50^ and osteocytes^51^. The set of genes used for mRNA expression profiling of osteoblasts, osteocytes, and bone lining cells by qRT-PCR was based on published data^52^. As shown in Fig. 4B and Suppl. Fig. 9, bone lining cells were characterized as *tissue nonspecific alkaline phosphatase* (*Alpl*)-, *Runx2*-, *Stanniocalcin2* (*Stc2*)-, *Reelin* (*Reln*)-, *Podoplanin* (*Pdpl*)-, *osteopontin* (*Spp1*)-, *phosphate*-*regulating gene with homologies to endopeptidases on the X*-*chromosome* (*Phex*), *parathyroid hormone receptor*-*1* (*PTHR1*)-, *RANKL*-, *dentin matrix protein*-*1* (*Dmp1*)-, and *sclerostin* (*Sost*)-expressing cells. Compared with osteoblasts, bone lining cells showed lower mRNA expression of *collagen 1a1* (*Col1a1*), *collagen 15a1* (*Col15a1*), *Runx2*, *osteonectin* (*Sparc*), *Keratocan* (*Kera*), *RANKL*, *OPG*, and *osteocalcin* (*Bglap*), but consistently higher expression of *Podoplanin*, *Phex*, *PTHR1*, and *sclerostin*. Compared with osteocytes, mRNA expression of *Phex*, *Dmp1*, *Stanniocalcin2*, *osteopontin*, and *osteocalcin* was lower, and expression of *Runx2*, *Podoplanin*, and *Alpl* was higher in bone lining cells. Overall, these results define bone lining cells as a distinct cellular compartment, and place them in between osteoblasts and osteocytes regarding their mRNA expression pattern. The genes encoding for CD34 and CD45R could not be amplified in LCM preparations of osteoblasts, osteocytes, and bone lining cells (data not shown), excluding a potential contamination with CD34-positive endothelial or CD45R-positive hematopoietic cells. Notably, *Sost* mRNA expression was also detected in osteoblasts and bone lining cells, a finding that was further confirmed at the protein level by immunohistochemistry on bone cryosections (Suppl. Fig. 10).

We next asked the question whether bone lining cells express ERα and β. We found *ERα*, and 5- to 10-fold lower *ER*β, mRNA expression in osteocytes, bone lining cells, and osteoblasts, suggesting that estrogen can target each of those cell types (Fig. 4C). However, mRNA expression of *ER*α and β was about 50% lower in bone lining cells, relative to osteoblasts (Fig. 4C). In line with the low immunoreactivity for RANKL protein in osteocytes by immunostaining, we found lower levels of *RANKL* mRNA expression in osteocytes compared with osteoblasts and bone lining cells in SHAM controls (Fig. 4D). Ovariectomy was associated with a 3-fold upregulation of *RANKL* mRNA expression in bone lining cells, which was corrected to SHAM control levels by estradiol replacement therapy of OVX mice (Fig. 4D). A similar trend was detected in osteoblasts and osteocytes, with a non-significant increase in RANKL expression in OVX mice. This led us to conclude that in line with our immunohistochemical data, estrogen primarily regulates RANKL expression in bone lining cells *in vivo*. In addition, mRNA analysis uncovered that estrogen deficiency not only upregulates the surface extent of RANKL-expressing bone lining cells as measured by immunohistochemistry, but also the relative expression of *RANKL* mRNA within the bone lining cell compartment.

## Discussion

**Figure 5:**
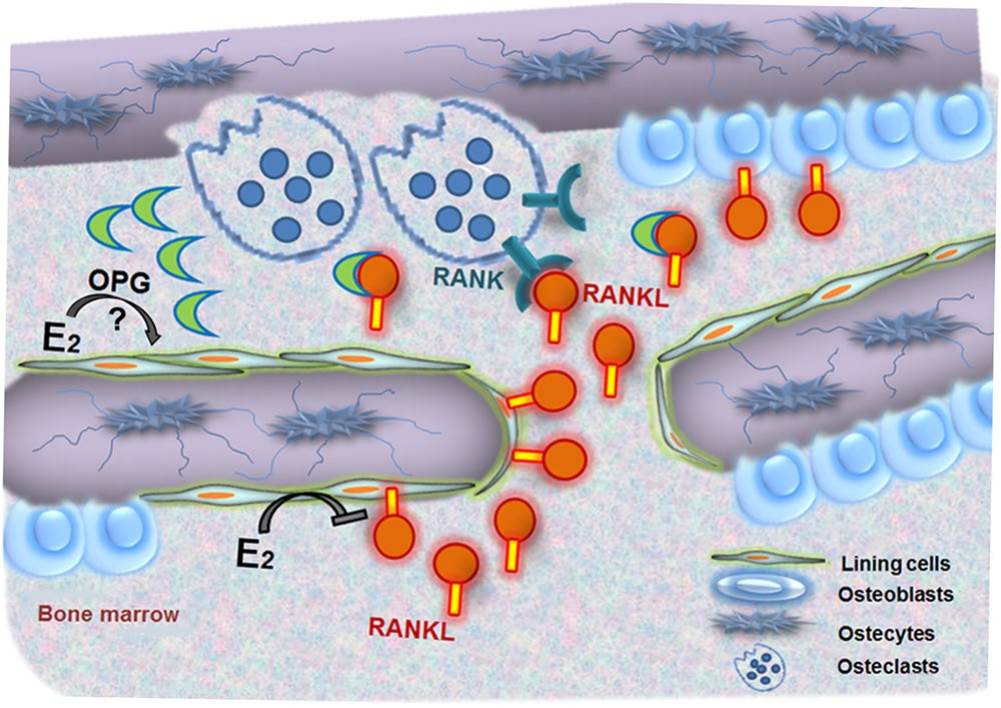
Model of how estrogen regulates osteoclastic bone resorption. Estradiol (E2) primarily suppresses RANKL expression in bone lining cells. Membrane-bound or soluble RANKL interacts with RANK expressed on mature osteoclasts and osteoclast precursors to stimulate bone resorption. The decoy receptor osteoprotegerin (OPG) binds and, thus, biologically inactivates membrane-bound and soluble RANKL. Estrogen deficiency reduces the suppression of RANKL expression by bone lining cells, leading to increased osteoclastic bone resorption. Estradiol may also regulate OPG expression in bone lining cells.

We undertook this study to decipher the cellular mechanisms by which estrogen deficiency upregulates bone turnover and causes bone loss in rats and mice. The evidence garnered from a number of novel experimental approaches indicated that estrogen regulates bone turnover primarily by regulating RANKL expression in bone lining cells in an ERα dependent manner. In contrast, ERα-mediated signaling in hematopoietic cells and RANKL derived from hematopoietic cellular sources did not impact on the physiological bone turnover in OVX mice. This model is shown in Fig. 5.

Bone remodeling starts with the resorption of a given quantity of bone by osteoclasts. Because bone lining cells cover all resting bone surfaces, they have long been considered to control osteoclastic bone resorption^53^. Bone lining cells were discussed to initiate bone resorption at a given site by removing the thin layer of osteoid covering quiescent bone surfaces^53^. In addition, bone lining cells are in contact with osteoblasts and underlying osteocytes via gap junctions^54^. Therefore, bone lining cells are ideally positioned to serve as gatekeepers of hormonally controlled bone resorption. Our data suggest that the increase in bone resorption observed in states of estrogen deficiency is mainly caused by lack of ERα-mediated suppression of RANKL expression in bone lining cells. A limitation of the current study is that we focused on estrogen deficiency-induced bone loss. However, estrogen is not the only endocrine regulator of bone remodeling. It is currently unknown whether the effects of other hormones such as androgens or parathyroid hormone on bone remodeling are also mediated by bone lining cells.

The conclusion of our study is supported by the study by Henning and coworkers, who also used a bone marrow reconstitution model with selective deletion of ERα^55^. The authors also reached the conclusion that the effect of estrogen on bone requires a functional ERα in non-hematopoietic, mesenchymal cells. An important difference to our study is the fact that those authors reported that the skeletal effects of estrogen in mice were enhanced via ERα–mediated effects in hematopoietic cells. The reason for this discrepancy may be related to differences in experimental design, and/or to different dosing of estrogen. Henning and coworkers ovariectomized 10-week-old mice 2 weeks before BMT and started estrogen replacement 2 weeks after BMT. Thereby the mice were deprived of estrogen for 4 weeks before estrogen treatment was initiated. In addition, Henning and coworkers supplemented estradiol via subcutaneous slow release pellets, resulting in pronounced bone anabolic effects. In contrast, we used near-physiological estradiol doses given by daily injections. It is known that subcutaneous slow release pellets show non-linear release kinetics^56^. Despite these discrepancies both studies provide strong evidence that estrogen regulates bone turnover primarily via acting on mesenchymal cells.

Lethal irradiation and bone marrow transplantation is frequently associated with bone loss in human patients (reviewed in ref. 57). In lethally irradiated female wild-type mice reconstituted with WT bone marrow and supplemented with physiological doses of estrogen, we failed to observe irradiation-induced bone loss at the distal femur and the spine. Hence, the present study suggests that post-transplantation bone loss in female patients receiving hormone replacement therapy is not related to irradiation *per se*, but rather a consequence of drug therapy to mitigate graft-versus-host disease. However, the small but significant reduction in vertebral cancellous bone volume as measured by µCT at the spine of estrogen-deplete OVX mice supports the notion that irradiation could aggravate bone loss in estrogen deficient patients.

During recent years, Rowe and coworkers have done pioneering work to establish the tape technology for bone cryosections, permitting high quality cryosections of undecalcified bone^47^. We used these high quality cryosections of undecalcified bone for laser capture microdissection, permitting sensitive *in situ* mRNA profiling of osteoblasts, osteocytes, and bone lining cells. In contrast to a recently published and independently developed LCM method based on labeling of bone forming surfaces by *in vivo* fluorochrome labeling in rats^58^, our method is based on morphological criteria to define the different cell types. Besides species differences, these methodological differences may explain the discrepancies between the cell type-specific mRNA expression profiles yielded by the different technologies. For example, Nioi and coworkers found only negligible expression of *sclerostin*, *Mepe* and *Dmp1* mRNA in osteoblasts and bone lining cells relative to osteocytes, and similar expression of *collagen 1a1* mRNA in osteoblasts and osteocytes, findings which are in clear contrast to our data. The advantage of our morphology-based LCM technology is that it allows specific sampling of the different cell types and minimizes the contamination of LCM-harvested bone surface cells with bone marrow cells. The brief staining necessary for our method does not interfere with RNA quality.

Our findings may also have major implications for experiments with conditional knockout mice, using Cre-loxP technology to delete genes in a cell-specific manner in bone. The *in situ* mRNA expression profiling data presented in this report showed for the first time that expression of genes such as *Phex*, *DMP1*, and *sclerostin* is not specific for osteocytes, as osteoblasts and bone lining cells also express moderate-to-low levels of these mRNAs. Hence, our data cast doubt whether a clear distinction between osteoblasts, osteocytes, and bone lining cells is possible by a conditional knockout approach on the basis of the currently available Cre driver mice. This issue may also account for the discrepancy between our data and those of Fujiwara and coworkers^27^, who recently reported that mice lacking RANKL in osteocytes are protected from estrogen deficiency-induced bone loss. To delete RANKL specifically in osteocytes, the latter authors used Dmp1-Cre mice. However, there is evidence from lineage tracing experiments employing Dmp1-Cre-ERT2 mice that the Dmp1 promoter is active in osteoblasts and their descendants^59, 60^, corroborating our data.

The operating model presented in this report, namely that estrogen primarily targets bone lining cells rather than osteocytes, osteoclasts, or immune cells for the regulation of physiological bone turnover, is also supported by an entirely distinct line of evidence: By using transgenic mice with a luciferase reporter gene under the control of three estrogen-responsive elements, it was reported earlier that estrogen treatment increased luciferase expression determined by immunohistochemistry in hypertrophic growth plate chondrocytes, megakaryocytes, osteoblasts, bone lining cells, and a subpopulation of osteocytes. In contrast, the majority of osteocytes and mature lymphocytes failed to respond to estrogen treatment with increased reporter gene expression^61^.

There is good evidence that osteocytes are quantitatively the main source of RANKL required for osteoclast formation in bone^24–26^, and our study does not challenge this view. However, our findings are at variance with the notion that osteocytes are exclusively orchestrating the action of estrogen on bone remodeling^27^. Although matrix-embedded osteocytes may be the main cell type mediating the detrimental effects of mechanical unloading on bone mass through increased secretion of RANKL^25^, our report suggests that a so far neglected cell type of the osteoblast lineage situated at the bone surface, namely bone lining cells, plays a key role in mediating the effects of estrogen deficiency on bone. Bone lining cells may actually work as cellular integrators of hormonal signals from the extracellular milieu, and of mechanical signals originating from the osteocyte network within bone.

## Methods

### Animals

All experimental procedures involving animals were conducted in accordance with prevailing guidelines for animal care and welfare, and were approved by the Ethical Committees of the University of Veterinary Medicine Vienna and of the local government authorities. All mice for this study had been backcrossed to C57BL/6 genetic background for more than 7 generations to prevent graft-versus-host disease post-transplantation. huRANKL-KI and αERKO-mice were genotyped from tail biopsies by PCR analysis of genomic DNA as described^43, 62^. The mice were kept at 22–24 °C with a 12 h/12 h light/dark cycle, and had free access to tap water and normal mouse chow (Ssniff Spezialdiäten GmbH, Soest, Germany). All experiments were performed on 16-week-old female offspring of heterozygous matings. At the age of 16 weeks female wild-type, huRANKL-KI and αERKO mice were lethally irradiated and bone marrow-transplanted (see below). After lethal irradiation, all mice received estrogen replacement therapy (10 µg 17 ß-estradiol (E2, Sigma-Aldrich)/kg 5 times per week in benzylbenzoate/ricinus oil 4 + 1 v/v, B/R). After a recovery phase of 4 weeks all irradiated mice were ovariectomized under anesthesia with isofluorane, and subsequently received either B/R vehicle or 10 µg E2/kg 5 times per week in B/R, or physiological saline or 10 mg/kg AMG161 (Amgen, Thousand Oaks, CA) dissolved in physiological saline twice weekly. Animals killed prior to irradiation or ovariectomy served as baseline controls. All animals were subcutaneously injected with calcein (Sigma-Aldrich, 20 mg/kg) on days 8 and 6 before necropsy. Urine was collected from all mice in metabolic cages one day prior to necropsy. Following double calcein labeling, all mice were euthanized 4 weeks after ovariectomy at 24 weeks of age by exsanguination from the abdominal V. cava under anaesthesia with ketamine/xylazine (67/7 mg/kg i.p.). Three-week-old *Rankl* ^∆/∆^ mice on C57BL/6 genetic background were kindly provided by Melita Ticevic and Josef M. Penninger, IMBA, Vienna, Austria.

In some experiments 6-month-old female Fischer 344 rats were ovariectomized or sham-operated under anesthesia with isofluorane. Thereafter, the rats were treated with vehicle or 2.5 µg/kg estradiol dissolved in B/R. Two weeks post-OVX, all rats were killed by exsanguination from the abdominal aorta under anesthesia with ketamine/xylazine (50/10 mg/kg i.p.).

### Lethal irradiation and bone marrow transplantation (BMT)

Wild-type, huRANKL-KI and αERKO mice received a single dose of 10 Gray using a linear accelerator (6 MV, Primus, Siemens) available at the University of Veterinary Medicine Vienna. In preliminary experiments, we tested single doses of 9–12 Gy. Four hours after irradiation, recipients received intravenously via tail vein 4 × 10^6^ unfractionated bone marrow cells (BMC) isolated from femurs, tibias and humeri of donor animals. Antibiosis was continued for one week post-irradiation. Mice were weighed every day for at least 10 days after BMT to control for health status.

### Isolation of BMC for transplantation

BMC were isolated from femurs, tibias and humeri of donor animals. Bones of donors were dissected directly after the mice were killed by cervical dislocation. Bones were cleaned from residual soft tissue, and cut in half. The bones were flushed with PBS (pH 7.4, Lonza) to harvest the BMC. Subsequently, BMC were centrifuged at 4 °C at 300 g for 5 min, resuspended in 1 ml of PBS, counted, and stored at 4 °C until transplantation.

### FACS analysis

To analyze the degree of chimerism in the bone marrow of hPLAP-transplanted WT mice after lethal irradiation with different irradiation dosages, FACS analysis of BMC was performed, 4 weeks post-transplantation. BMC were isolated as described above, and stained with a mouse anti-hPLAP monoclonal antibody (H17E2, Bio-Rad AbD Serotec). Before staining of cells, this antibody was directly labeled with allophycocyanine (APC, APC conjugation kit, AbD Serotec). 10 µl of the Ab working dilution (10 µg/ml) were used to label 5 × 10^5^ cells. Cells were incubated for 30 minutes at 4 °C in the dark. Thereafter, the cells were washed two times with 1 ml PBS (1200 U/min, 3 min, 4 °C). By mixing BMC of WT and hPLAP-tg mice, a standard curve was generated and stained in parallel to determine the degree of bone marrow chimerism of hPLAP-transplanted WT mice. BM subpopulations were stained using antibodies (all purchased from BD Biosciences) directed against Gr1 (Ly6G/Ly6C, RB6-8C5), Ter119 (Ly-76, Ter119), CD19 (1D3), CD3 (CD3ε, 145-2C11) and Mac1 (CD11b, M1/70). Samples were analyzed on a FACSCanto II flow cytometer (Becton-Dickinson) using FACSDiva software.

### Isolation and culture of MSCs

MSCs were isolated using a modification (additional collagenase digestion and Ficoll gradient centrifugation steps) of the protocol described by Farrell *et al*.^63^. Immediately before tissue collection the animals were euthanized by cervical dislocation. After cleaning, humeri, femurs and tibias were cut at both ends and incubated in sterile modified Eagle’s medium with Earle’s Salts and L-glutamine (MEM; PAA Laboratories) containing 2.5 mg/mL collagenase type II (GibcoTM, Invitrogen) for 2 h at 37 °C, 5% CO_2_, and 3% O_2_. Thereafter, the BMC were flushed out with 5 mL of complete MEM supplemented with 20% heat-inactivated fetal calf serum (FCS; PAA Laboratories) and 1% penicillin/streptomycin (PAA Laboratories). Cells were resuspended in fresh complete culture medium. Thereafter, the mononuclear cell fraction was collected after density gradient centrifugation using Ficoll-PaqueTM (500 g, 30 min; GE Healthcare), washed, and finally resuspended in complete MEM with 1% ascorbic acid and 0.12% FGF2. The mononuclear cell fraction was plated in T25 cell culture flasks (PAA Laboratories GmbH). Upon reaching 80% confluency, cells were passaged, replated and were subcultured up to passage 3. The medium was changed twice weekly during culture and the cells were maintained at 37 °C, 5% CO_2_, and 3% O_2_.

### hPLAP expression

Histochemical staining was performed to evaluate hPLAP expression as described^40^. In brief, cells were fixed in ice-cold acetone–methanol (30:70, v/v) for 5 min. Following two washing steps using PBS, endogenous heat-labile alkaline phosphatases were inactivated by incubation in TMN substrate buffer (0.1 M Tris- HCl, pH 9.5 containing 0.1 M NaCl and 5 mM MgCl_2_) at 60 °C for 30 min. Thereafter, staining for heat-stable hPLAP was performed at RT for at least 3 h by using fresh TMN buffer containing 0.175 mg/mL of 5-bromo-4-chloro-3-indolyl phosphate (BCIP; Sigma-Aldrich) and 0.45 mg/mL of nitrotetrazolium blue chloride (NBT; Sigma-Aldrich)^40^. After two washing steps, samples were evaluated under a stereo microscope (Stemi SV6; Zeiss) or an inverted light microscope (Axiovert 25; Zeiss). Each sample was stained at least in triplicate. MSC isolated from WT mice served as negative control.

### Clinical chemistry

Total urinary deoxypyridinoline was measured by EIA (MicroVue DPD EIA kit, Quidel), and expressed per urinary creatinine concentration. Urinary creatinine was analyzed with a Cobas c111 autoanalyzer (Roche Diagnostics).

### Bone histology & bone histomorphometry

Bones for histomorphometric analysis were fixed in 4% paraformaldehyde (PFA) at 4 °C for 24 h, and embedded in a modified methylmethacrylate embedding mixture suitable for histochemistry and immunohistochemistry^64^. Cancellous bone histomorphometry in the distal femoral metaphysis was performed using OsteoMeasure 3.0 (OsteoMetrics) software as described^44^.

### pQCT and µCT measurements

Bone mineral density (BMD) of the right femur and the 4th lumbar vertebra was measured as described^65, 66^ by peripheral quantitative computed tomography (pQCT) using an XCT Research M + pQCT machine (Stratec Medizintechnik) with a voxel size of 0.070 mm. In brief, three 0.2-mm-thick slices in the distal femoral metaphysis located 0.5, 1, and 1.5 mm proximal to the articular surface of the knee joint, and one slice in the mid-diaphysis of the femur were measured. In the 4th lumbar vertebrae, three 0.2-mm-thick slices were measured in the vertebral bodies, located in a mid-transversal plane and 2 planes located 0.8 mm cranially and caudally of the mid plane. BMD values of the distal femoral metaphysis and of the 4th lumbar vertebra were calculated as the mean over 3 slices. Thresholds of 450 mg/cm³ and 600 mg/cm³ were used or the calculation of trabecular and cortical BMD, respectively.

Quantitative microcomputed tomography (µCT) using a Scanco µCT 35 machine (Scanco) was performed to assess bone microarchitecture of right femurs and L4 lumbar vertebrae as described^66^. A 0.5 mm aluminum filter was used to improve image quality. In the femoral shaft, a segment of 805 µm in the middle of the femur was scanned (115 slices, isotropic voxel size of 7.0 µm). Voxels with intensities greater than 9174 (corresponding to a linear attenuation of µ = 2.24/cm) were taken to be bone. In the distal femoral metaphysis, we scanned an approximately 3000-µm large segment (462 cross-sectional slices, isotropic voxel size of 6.0 µm). A segment of 1020 µm thickness (170 slices) at a distance of 840 µm from the distal growth plate was used for evaluation of the distal femoral metaphysis. Voxels with intensities greater than 6553 (corresponding to a linear attenuation of µ = 1.60/cm) were taken to be bone in this region. In L4 lumbar vertebrae, we scanned a segment of 812 µm in the caudal part of the vertebral bodies (116 slices, isotropic voxel size of 7.0 µm). Voxels with intensities greater than 6553 (corresponding to a linear attenuation of µ = 1.60/cm) were taken to be bone in the vertebrae. µCT images were reconstructed in either 2048 × 2048 or 1024 × 1024 pixel matrices using a standard convolution-back projection procedure, and the resulting gray-scale images were processed using a 3D Gaussian filter with σ = 0.5 to remove noise. Automatic contouring (inner and outer boundary of cortical bone) and morphometric evaluation was done by the inbuilt Scanco software, using assumption-free methods.

### Immunohistochemistry

Four-µm-thick cryosections of bone samples snap-frozen in liquid nitrogen with OCT compound (Sakura Finetek, Zoeterwoude, Netherlands) were cut on a cryotome (Leica Kryostat 1720), using the cryotape method as described^47^. Sections were fixed in 100% methanol at −20 °C for 2 min, and rinsed in PBS 3 × 5 min. Nonspecific binding was minimized by pre-incubation with 10% normal goat serum (Vector) containing 0.02% Triton X-100 (Sigma-Aldrich) for 60 min at RT. Incubation with rabbit anti-RANKL (Santa Cruz FL317), diluted 1:200 in blocking solution, was carried out at 4 °C overnight. For negative controls, the primary antibody was omitted. Endogenous peroxidase activity was inhibited by 0.3% H_2_O_2_ in PBS for 15 min. After washing in PBS 3 × 5 min, the sections were incubated with biotinylated goat anti-rabbit secondary antibody (1:200, Vector) for 60 min. at RT, followed by peroxidase-labeled streptavidin (Invitrogen) for 30 min at RT. DAB staining (Invitrogen) was employed for antibody detection. Subsequently, the sections were counterstained with hematoxylin, and mounted in glycerol gelatin. The relative bone surface covered by RANKL-positive osteoblasts and bone lining cells as well as the percentage of RANKL-positive osteocytes was quantified using OsteoMeasure 3.0 (OsteoMetrics) software. Anti-RANKL immunohistochemistry in 5-µm-thick paraffin sections of 40% ethanol-fixed spleens was performed in an identical fashion, with the modifications that the primary antibody (Santa Cruz FL317) was diluted 1:100, and the secondary antibody 1:400. In addition, bound antibody was detected by AEC staining (3-amino-9-ethylcarbazole, Invitrogen).

Immunohistochemistry using the R&D anti-RANKL antibody (RD AF 462) was performed on methanol-fixed 5-µm-thick bone cryosections by pre-incubation with 5% BSA (Sigma) in PBS for 90 min at RT, followed by incubation with goat anti-RANKL, 5 µg/mL in blocking solution, at 4 °C for 5 days. For negative controls, the primary antibody was omitted. Endogenous peroxidase activity was inhibited by 0.5% H_2_O_2_ in PBS for 15 min, and the sections were incubated with biotinylated rabbit anti-goat IgG (H + L), F(ab′)2 fragment, (SAB3700311, Sigma) (7.5 µg/mL) for 60 min at RT, followed by incubation with peroxidase-labeled streptavidin (Invitrogen) for 30 min at RT, DAB staining (Invitrogen), counterstaining with hematoxylin, and mounting in glycerol gelatin. SOST immunohistochemistry was carried out similarly by using R&D anti-SOST (RD AF 1589) primary antibody (5 µg/mL) and biotinylated rabbit anti-goat IgG (H + L), F(ab′)2 fragment, secondary antibody (7.5 µg/mL).

### Laser capture microdissection (LCM)

Four-µm-thick cryosections of bone samples snap-frozen in liquid nitrogen with OCT compound (Sakura Finetek, Zoeterwoude, Netherlands) were cut on a cryotome (Leica Kryostat 1720), using the cryotape method as described^47^. Cryosections were fixed in 70% ethanol for 30 s at RT, washed in RNase-free water (Sigma), stained for 30 s in HistoStain (Arcturus), washed in water, dehydrated using ethanol (70/96/100%), and cleared with xylene (30 s each). Thereafter, the sections were air-dried. Dried samples were transferred onto MMI Membrane Slides (Alibaba Group) for microdissection. Microdissection was performed using a Veritas LCM system (Arcturus) within a time frame of about one hour maximum. Osteoblasts, osteocytes, and bone lining cells in distal femoral cancellous bone were captured on CapSure Macro LCM Caps (Life Technologies) based on morphologic criteria. Osteoblasts were defined as mononuclear, cuboidal cells covering osteoid, bone lining cells as flat cells covering quiescent bone surfaces, and osteocytes as matrix-embedded cells. Since it was reported that cells forming the envelope around red bone marrow express osteogenic markers^67^, care was taken to distinguish between bone lining cells attached to the bone surface and bone marrow envelope cells (Fig. 4A). Cutting laser intensity was set to high power, while capture laser intensity and spot size were set to 220 mV/100 mW and 15–20 µm, respectively. Typically, 50–100 osteoblasts, osteocytes, and bone lining cells each were captured in 2–3 sections per sample, and provided sufficient RNA for qRT-PCR. Harvested cells were immediately lysed in 100 µl isolation buffer of the SPLIT RNA Extraction Kit (Lexogen), and stored at −80 °C until used.

### RNA isolation and quantitative RT-PCR

RNA was extracted using the SPLIT RNA Extraction Kit (Lexogen) according to the manufacturer’s protocol. RNA purity and quality was determined by 2100 Bioanalyzer (RNA 6000 Pico Chip, Agilent Technologies). Only RNA samples with RIN values greater than 5 were used for subsequent analysis. Typical RIN values were between 6 and 9. 100–200 ng of RNA was used for first-strand cDNA synthesis (High-Capacity cDNA Reverse Transcription Kit, Applied Biosystems). Quantitative RT-PCR was performed on a ViiA™ 7 real-time PCR System (Applied Biosystems) using 5x HotFIREPol EvaGreen™ qPCR Kit (Solis Biodyne). A melting curve analysis was done for all assays. Primer sequences are available on request. Efficiencies were examined by standard curve. Regulation of gene expression was calculated according to Pfaffl^68^. Expression of target genes was normalized to the expression of the housekeeping gene ornithine decarboxylase antizyme 1 (Oaz1).

### Statistical analyses

SPSS for Windows 17.0 was used for computing statistics. The data were analyzed by 1-way analysis of variance (ANOVA) followed by Student-Newman-Keuls (SNK) multiple comparison test. P values of less than 0.05 were considered significant. The data are presented as the mean ± SD.

### Data availability

All data generated or analyzed during this study are included in this published article (and its Supplementary Information files).

## Electronic supplementary material

Supplementary data
