# Supplementary material for: Estrogen Regulates Bone Turnover by Targeting RANKL Expression in Bone Lining Cells
Source: Sci Rep. 2017 Jul 25;7:6460. doi: 10.1038/s41598-017-06614-0 (PMC5527119; doi:10.1038/s41598-017-06614-0)
Supplement: Supplementary file 1 — Supplementary data [file 41598_2017_6614_MOESM1_ESM.pdf]

Supplementary material

Estrogen Regulates Bone Turnover by Targeting RANKL Expression in Bone Lining Cells

Carmen Streicher[1 ƚ], Alexandra Heyny[1 ƚ], Olena Andrukhova[1], Barbara Haigl[1], Svetlana Slavic[1], Christiane Schüler[1], Karoline Kollmann[1], Ingrid Kantner[1 ʃ], Veronika Sexl[1], Miriam Kleiter[2], Lorenz C. Hofbauer[3], Paul J. Kostenuik[4 ¶], and Reinhold G. Erben[1] *

*** Corresponding author**:
Reinhold G. Erben, M.D., D.V.M.
Institute of Physiology, Pathophysiology and Biophysics
Dept. of Biomedical Sciences
University of Veterinary Medicine Vienna
Veterinaerplatz 1, 1210 Vienna, Austria
Phone +43-1-250 77 4550, Fax +43-1-250 77 4599
E-mail Reinhold.Erben@vetmeduni.ac.at

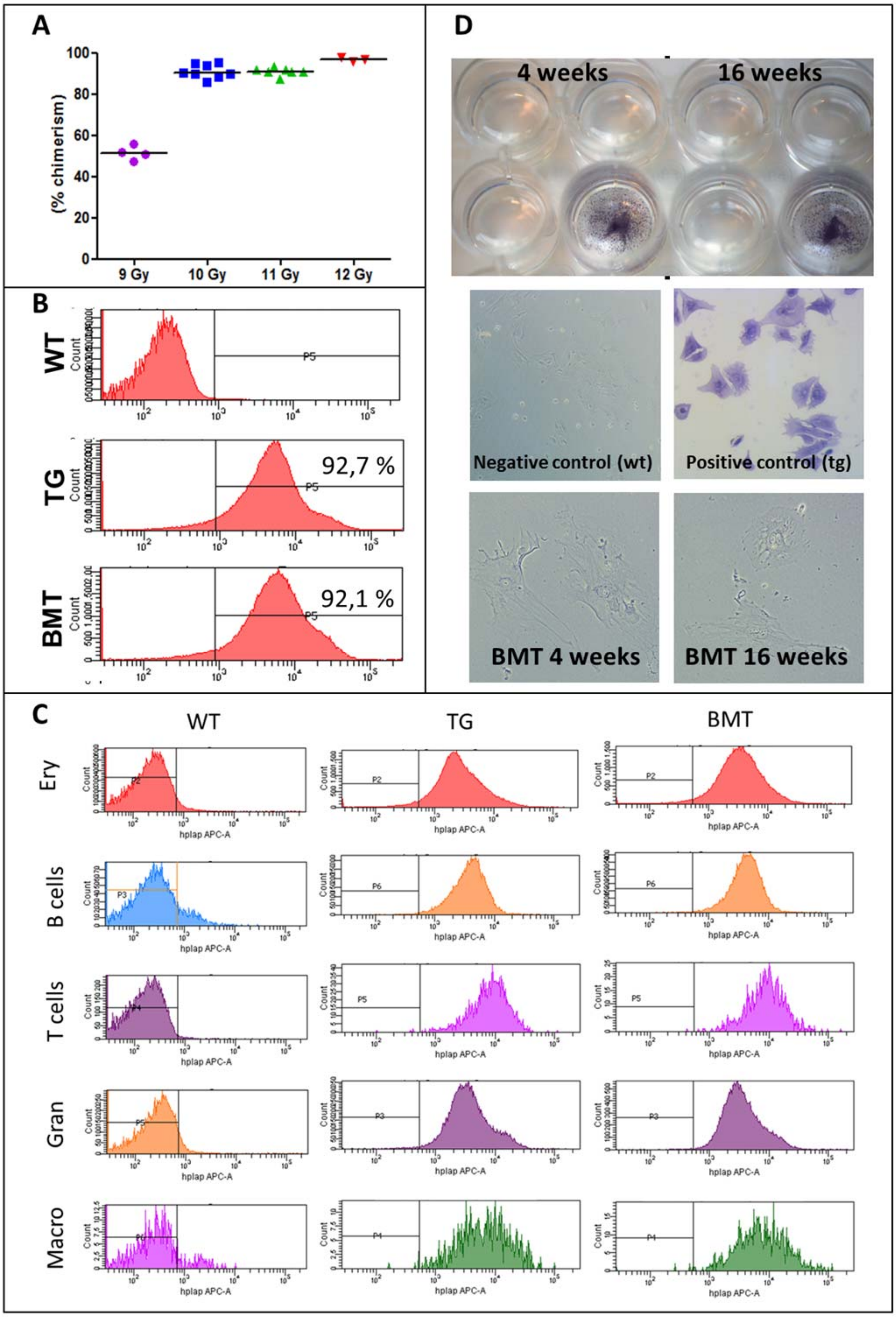

Supplementary Figure 1. Transplantation with unfractionated bone marrow after lethal irradiation. (A) Degree of chimerism after different lethal irradiation doses, 4 weeks after transplantation (BMT) of irradiated wild-type (WT) mice with bone marrow from hPLAP-tg mice. (B) FACS analysis of hPLAP expression in bone marrow cells of WT mice, hPLAP-tg (TG) mice, and WT mice transplanted with bone marrow from hPLAP-tg mice (BMT) after lethal irradiation with 10 Gy, 4 weeks post-transplantation. (C) Lineage-specific FACS analysis of bone marrow cells after lethal irradiation and bone marrow transplantation. Representative plots from FACS analysis of bone marrow cells isolated from wild-type (WT), hPLAP-tg (TG) and WT mice transplanted with bone marrow from hPLAP-tg mice (BMT) after lethal irradiation with 10 Gy, 4 weeks post-transplantation. Cells were co-stained with antibodies against hPLAP and lineage-specific antibodies for erythrocytes (Ery), B lymphocytes, T lymphocytes, granulocytes (Gran) and macrophages (Macro). (D) Expression of hPLAP in MSC isolated from WT mice (negative control), hPLAP-tg mice (positive control), and WT mice after reconstitution with hPLAP-tg bone marrow, 4 and 16 weeks post-transplantation. Upper panels show cell culture wells after heat inactivation and histochemical hPLAP staining, lower panels show high power images.

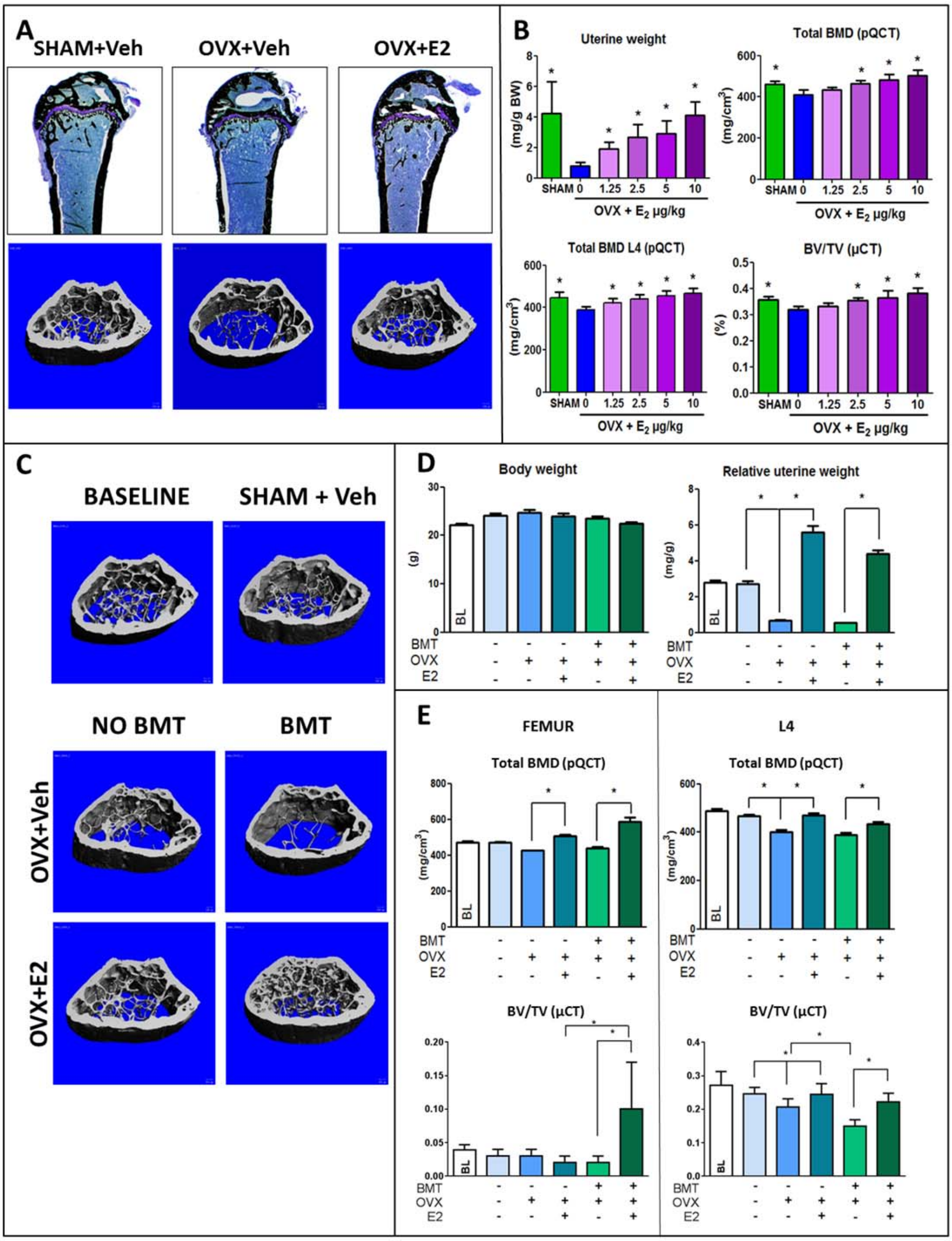

Supplementary Figure 2. Evaluation of physiological estradiol supplementation dosage in OVX mice and effect of irradiation on bone mass. (A) Five-µm-thick undecalcified sections of distal femurs stained with von Kossa/Mc Neal (upper panels), original magnification x25, and µCT images (lower panels), of the distal femoral metaphysis of vehicle-treated SHAM and vehicle-or estradiol (E2, 10µg/kg)-treated OVX C57BL/6 mice, 4 weeks post-OVX. (B) Uterine weight, total volumetric BMD measured by pQCT at the distal femoral metaphysis and at the L4 lumbar vertebral body, and bone volume (BV/TV) measured by µCT in the L4 lumbar vertebral body of vehicle-treated SHAM and vehicle-or E2-treated OVX C57BL/6 mice, 4 weeks post-OVX. Mice were ovariectomized at 12 weeks of age. Estradiol was given subcutaneously in benzylbenzoate/ricinus oil (1:5, v/v) five times a week. Each data point represents the mean ± SD of 7-8 mice each. * p> 0.05 by 1-way ANOVA followed by SNK test. (C) µCT images of the distal femoral metaphysis of baseline, sham-operated (SHAM), and vehicle- and E2-treated non-irradiated WT OVX mice, and irradiated WT mice reconstituted with WT bone marrow. (D) Body weight and relative uterine wet weight in baseline, sham-operated, as well as vehicle- and E2-treated non-irradiated and irradiated OVX WT mice reconstituted with WT bone marrow. (E) Total BMD and bone volume (BV/TV) of the distal femur and of the L4 vertebrae measured by pQCT and µCT, respectively, in baseline, sham-operated, as well as vehicle- and E2-treated non-irradiated and irradiated OVX WT mice reconstituted with WT bone marrow. Data in D and E represent mean ± SD of 8 – 10 animals each.* p<0.05 by one-way ANOVA followed by SNK test.

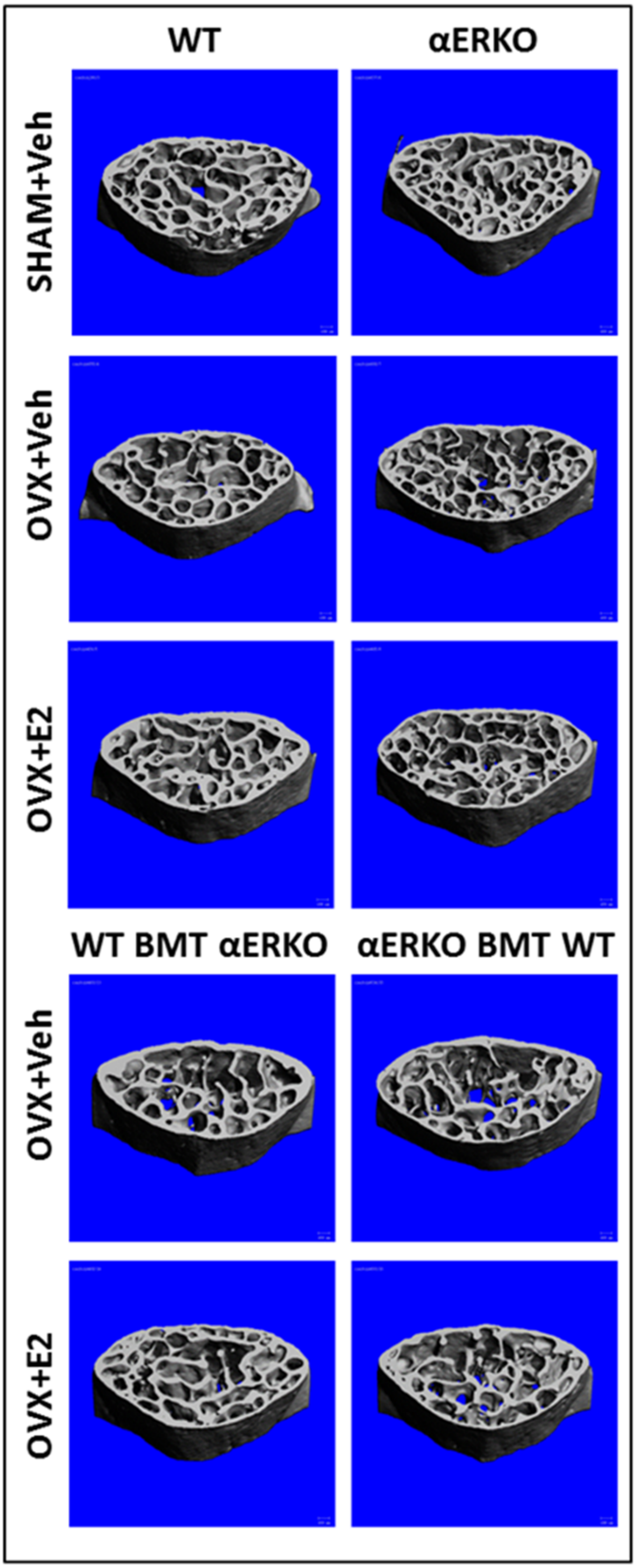

Supplementary Figure 3. µCT images of L4 vertebral bodies of non-irradiated (upper panels) SHAM and vehicle- or 17ß-estradiol (E2)-treated OVX WT and αERKO mice, and of irradiated (lower panels) vehicle- or estradiol-treated WT and αERKO mice transplanted (BMT) with unfractionated bone marrow cells from αERKO or WT mice, 4 weeks post-OVX.

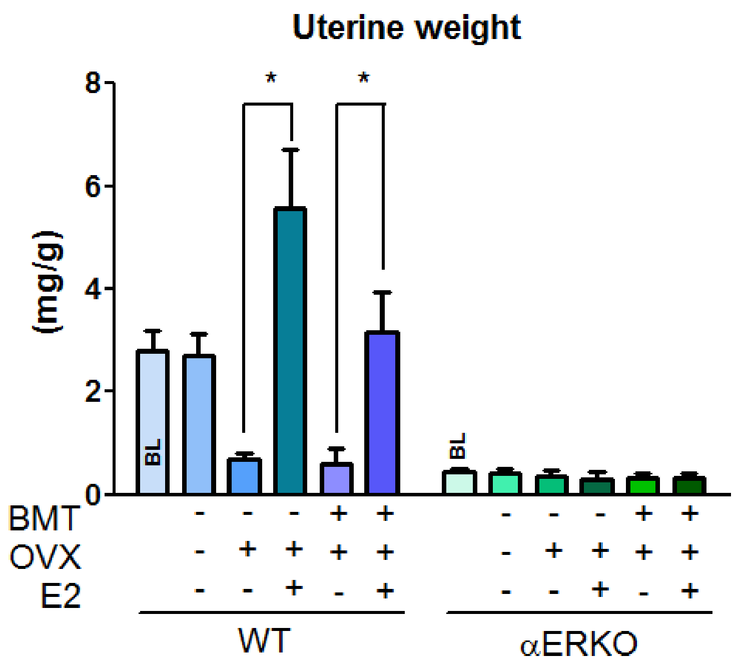

Supplementary Figure 4. Effects of selective deletion of estrogen receptor α in the mesenchymal or hematopoietic compartment on uterine weight. Uterine weight expressed per body weight in non-irradiated SHAM and vehicle- or 17ß-estradiol (E2)-treated OVX wild-type (WT) and αERKO mice, and in vehicle- or estradiol-treated WT and αERKO mice transplanted (BMT) with unfractionated bone marrow cells from αERKO or WT mice, 4 weeks post-OVX. Each data point represents the mean ± SD of 8-10 mice each. * $p < 0.05$ by 1-way ANOVA followed by SNK test. BL, baseline.

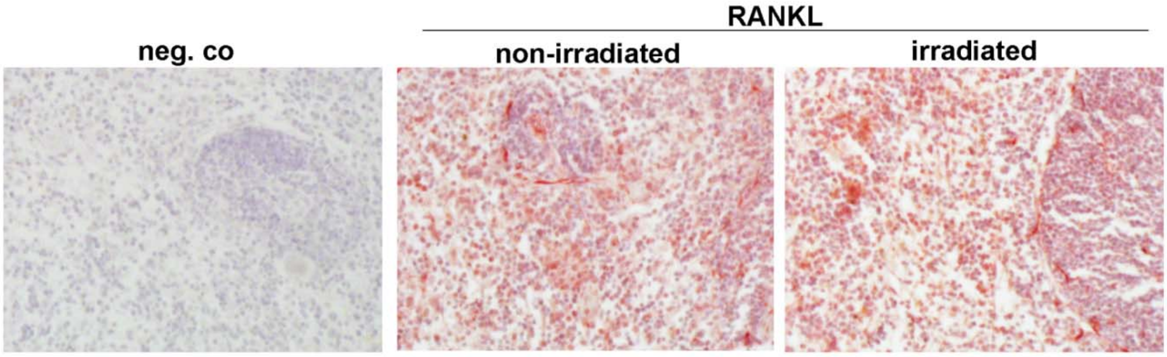

Supplementary Figure 5. Effect of irradiation on RANKL expression. Representative images of immunohistochemical anti-RANKL staining in 5-µm-thick paraffin sections of spleens from 4-month-old non-irradiated WT mice and irradiated WT mice, 4 weeks after irradiation and transplantation with bone marrow from hPLAP-tg mice. Sections were stained with anti-RANKL antibody FL-317 (Santa Cruz). Magnification 200x. Negative control comprises use of secondary antibody only.

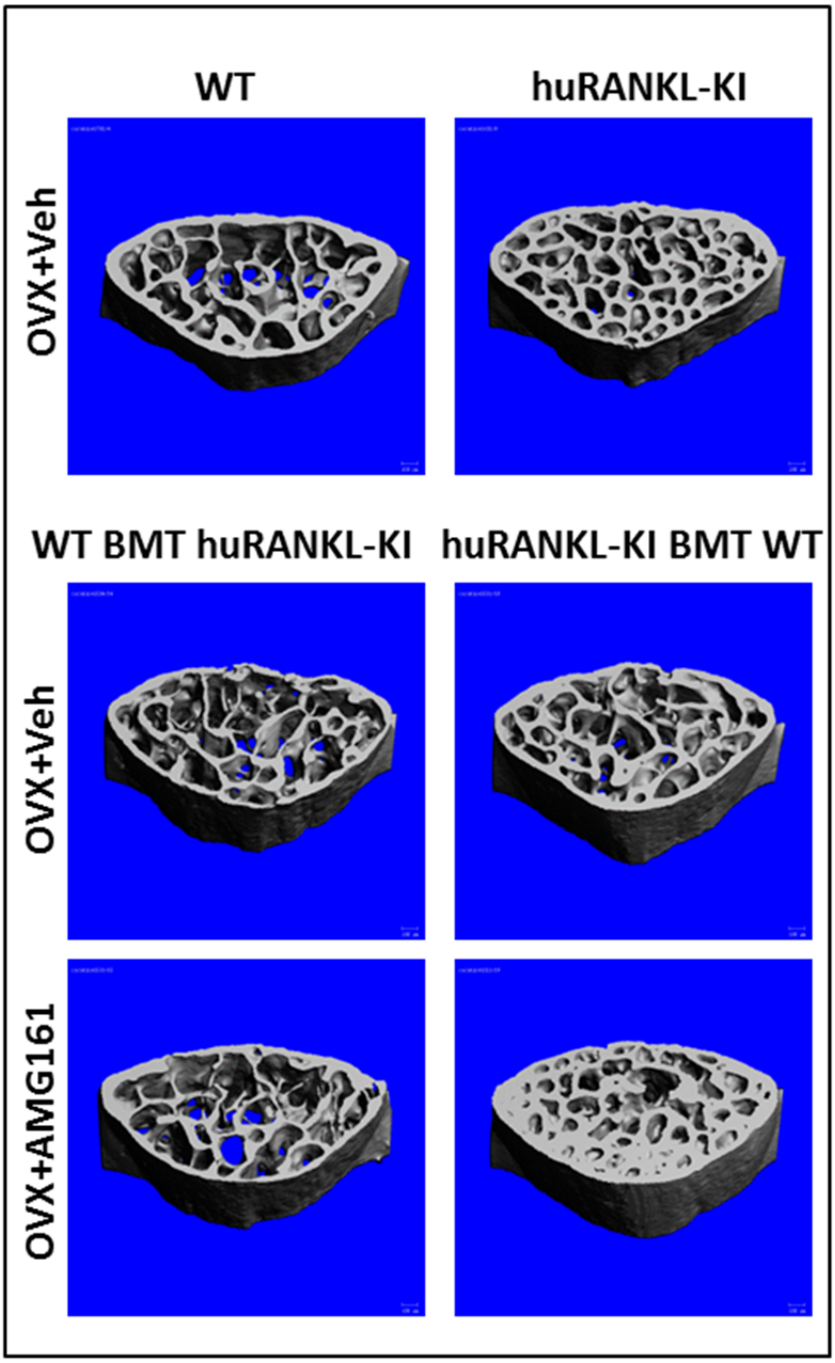

Supplementary Figure 6. μCT images of L4 vertebral bodies of non-irradiated vehicle-treated OVX WT and huRANKL-KI mice (upper panels), and of irradiated (lower panels) vehicle- or AMG161 (10 mg/kg twice weekly)-treated WT and huRANKL-KI mice transplanted (BMT) with unfractionated bone marrow cells from huRANKL-KI or WT mice, 4 weeks post-OVX.

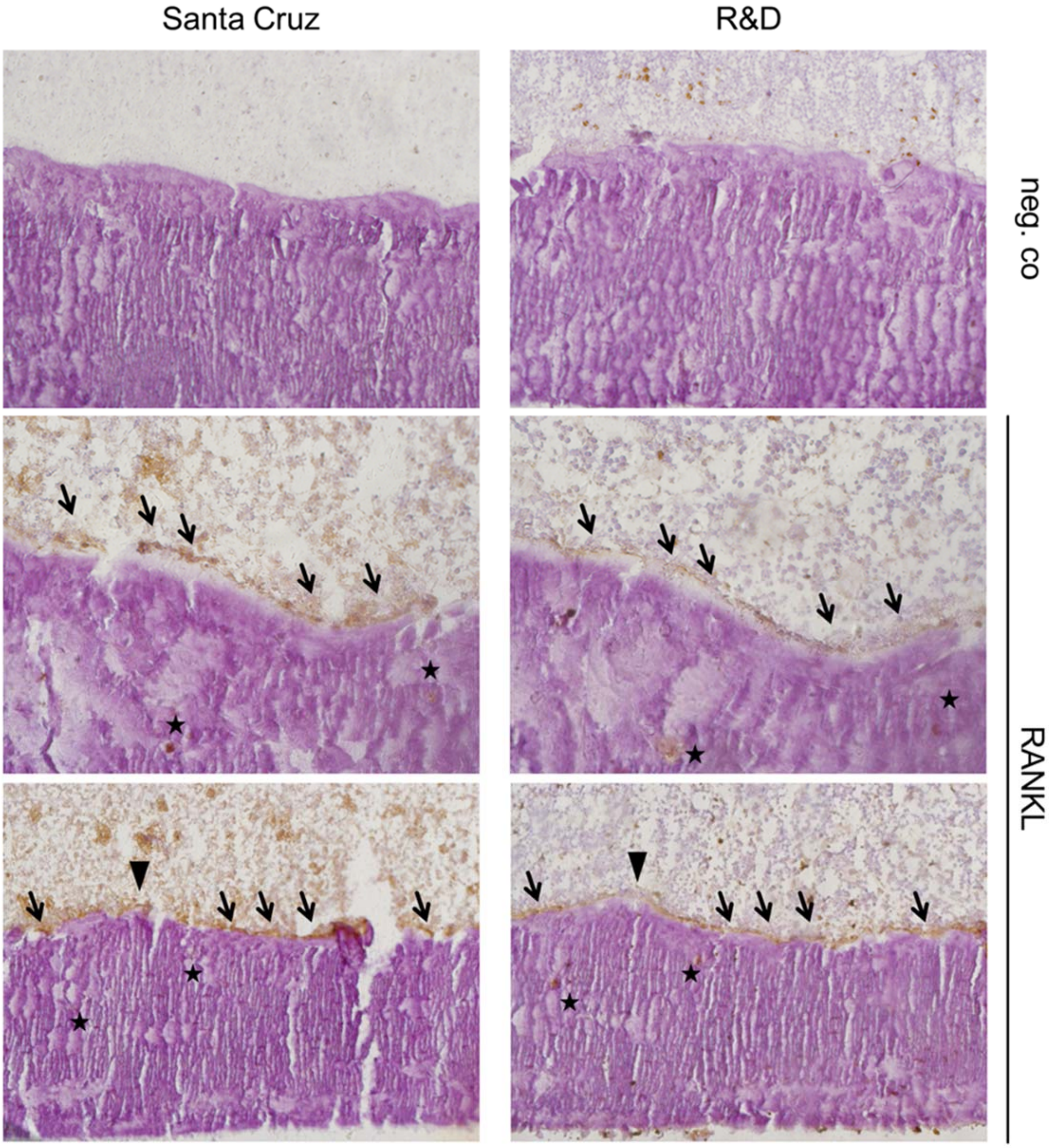

Supplementary Figure 7. Detection of RANKL expression by immunohistochemistry. Five-µm-thick undecalcified sections of distal femurs stained with anti-RANKL antibodies FL-317 (Santa Cruz) and RD AF 462 (R&D Systems). Comparison of RANKL expression detected by the Santa Cruz anti-RANKL antibody (left panels) and the R&D anti-RANKL antibody (right panels) in osteoblasts (arrows), osteocytes (asterisks), and lining cells (arrowheads). Negative control comprises use of secondary antibody only. Magnification 400x.

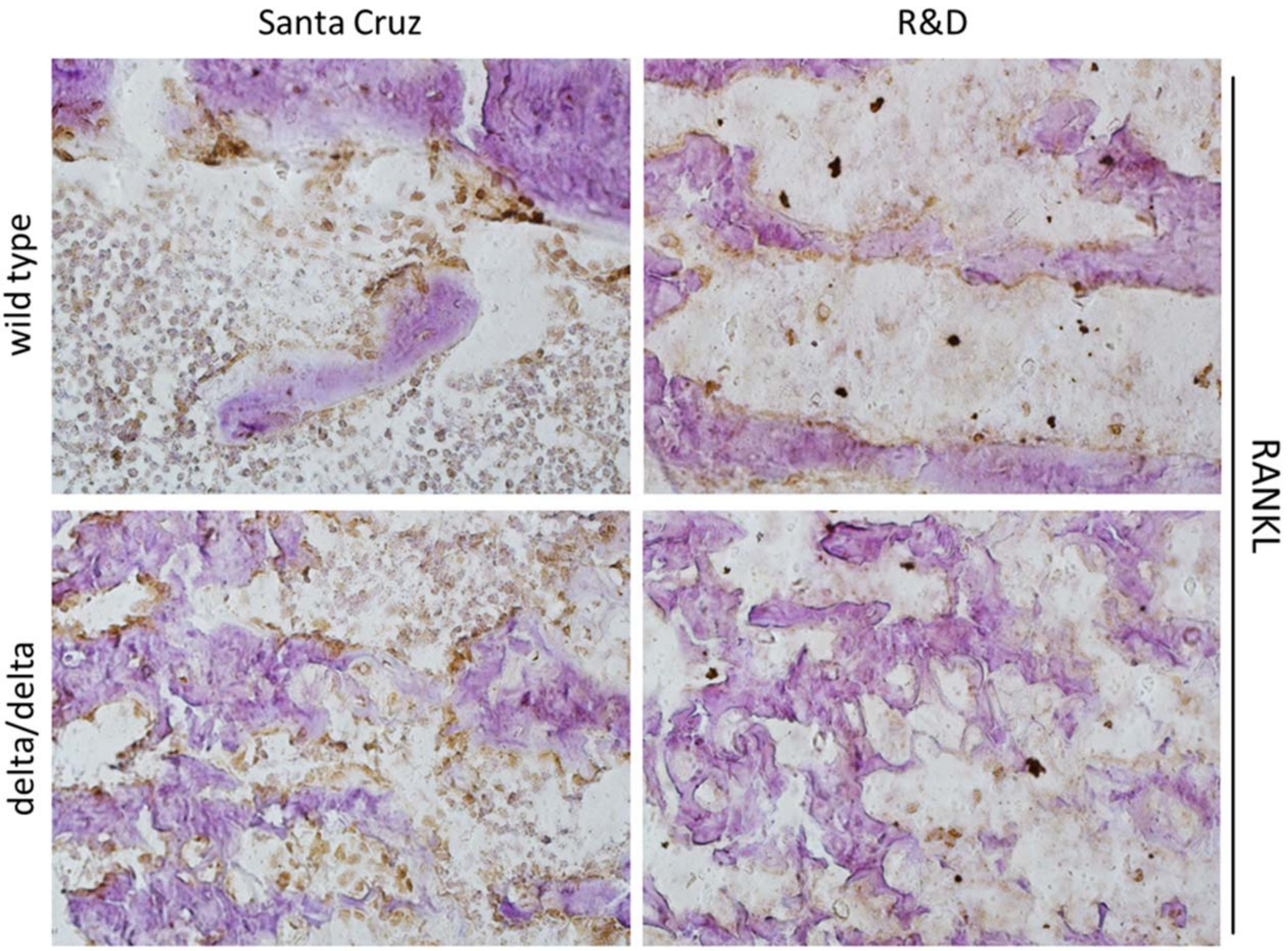

Supplementary Figure 8. Validation of anti-RANKL antibodies using wild-type and *Rankl*$^{\Delta/\Delta}$ mice. Five-µm-thick undecalcified cryosections of femurs of 3-week-old wild-type and *Rankl*$^{\Delta/\Delta}$ mice stained with anti-RANKL antibodies FL-317 (Santa Cruz) (left) and RD AF 462 (R&D Systems) (right). *Rankl*$^{\Delta/\Delta}$ mice are characterized by a deletion of the C-terminal half of the RANKL protein, and express a truncated RANKL protein consisting of the N-terminal ~130 aa, including the transmembrane domain. The Santa Cruz Ab was raised against aa 46-317, including the transmembrane domain, whereas the R&D Ab was raised against aa 72-316, excluding the transmembrane domain. The Santa Cruz Ab did not distinguish between wild-type and delta RANKL, suggesting that the transmembrane domain contains important immunogenic epitopes. In contrast, anti-RANKL staining using the R&D Ab was profoundly reduced in *Rankl*$^{\Delta/\Delta}$ compared with wild-type mice, verifying its specificity. Magnification 400x.

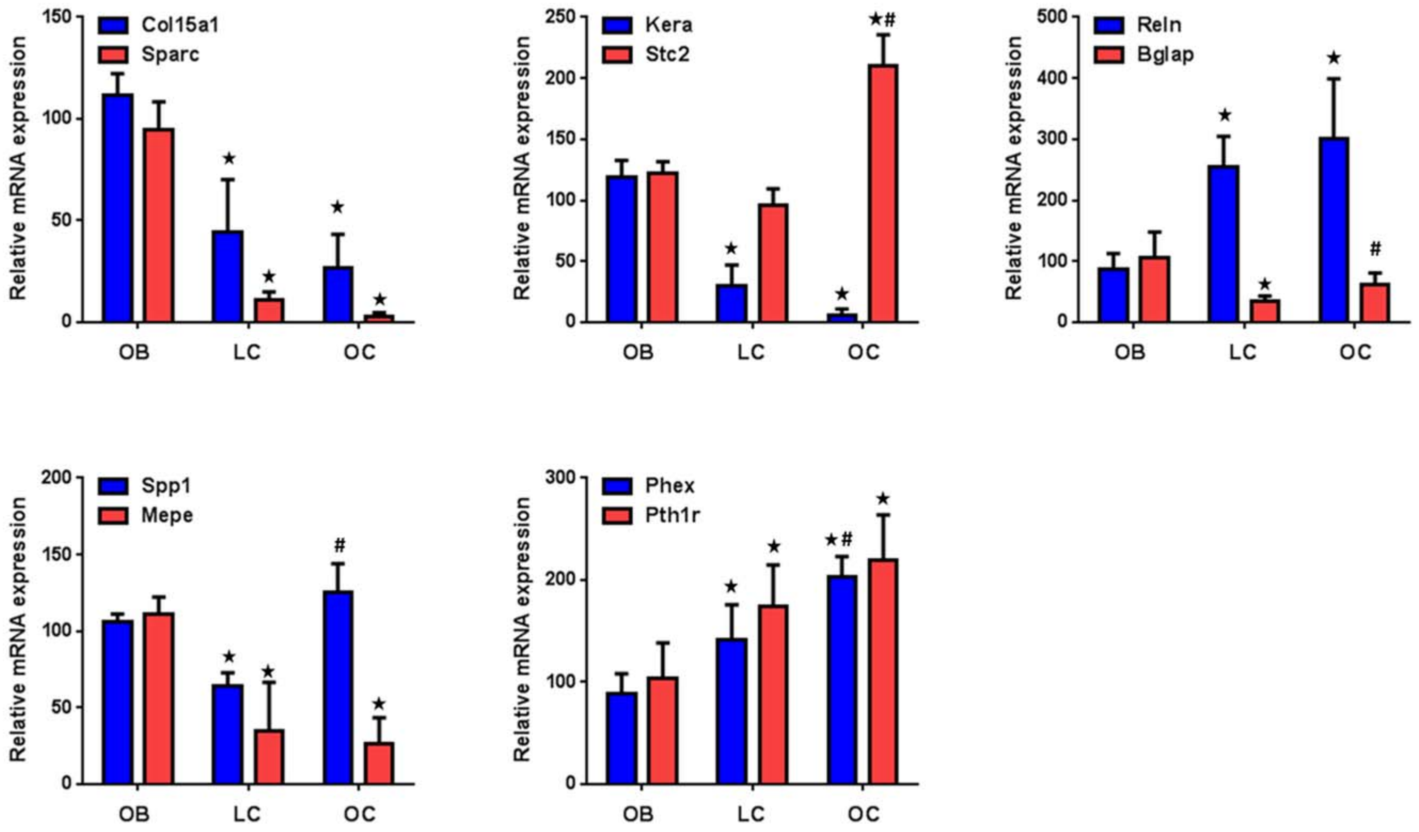

Supplementary Figure 9. Laser capture microdissection (LCM)-based *in situ* mRNA profiling of *collagen 15a1 (Col15a1), osteonectin (Sparc), Keratocan (Kera), Stanniocalcin2 (Stc2), reelin (Reln), osteocalcin (Bglap), osteopontin (Spp1, secreted phosphoprotein 1), matrix extracellular phosphoglycoprotein (Mepe), phosphate-regulating gene with homologies to endopeptidases on the X-chromosome* (*Phex*), *and parathyroid hormone receptor-1 (Pth1r)* mRNA by qRT-PCR on RNA isolated from distal femoral cancellous bone osteoblasts (OB), osteocytes (OC), and bone lining cells (LC) harvested by LCM in cryosections of undecalcified bones from WT mice. Data represent mean ± SD of 3 – 5 animals each. * denotes P<0.05 vs. osteoblasts, # denotes P<0.05 vs. lining cells by one-way ANOVA followed by SNK test.

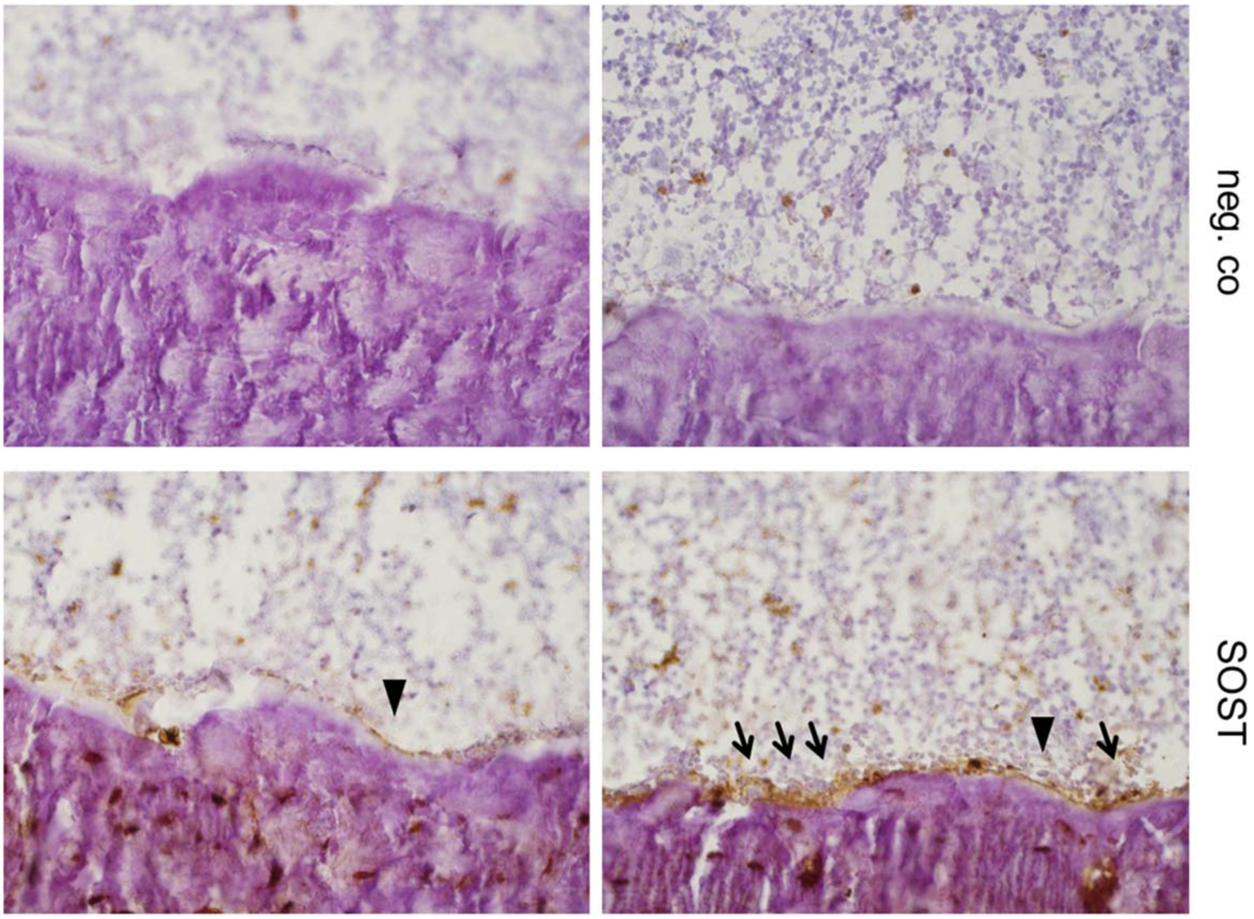

Supplementary Figure 10. Detection of sclerostin (SOST) expression in bone cells by immunohistochemistry. Five-µm-thick undecalcified cryosections of distal femurs stained with anti-mSOST antibody (RD AF 1589). SOST protein expression is evident in osteocytes, osteoblasts (arrows), and lining cells (arrowheads). Negative control comprises use of secondary antibody only. Magnification 400x.
